# Genomic and Phenotypic Analyses of *Acinetobacter baumannii* Isolates From Three Tertiary Care Hospitals in Thailand

**DOI:** 10.3389/fmicb.2020.00548

**Published:** 2020-04-06

**Authors:** Jessica Loraine, Eva Heinz, Rosesathorn Soontarach, Grace A. Blackwell, Richard A. Stabler, Supayang P. Voravuthikunchai, Potjanee Srimanote, Pattarachai Kiratisin, Nicholas R. Thomson, Peter W. Taylor

**Affiliations:** ^1^School of Pharmacy, University College London, London, United Kingdom; ^2^Liverpool School of Tropical Medicine, Liverpool, United Kingdom; ^3^Wellcome Sanger Institute, Hinxton, Cambridge, United Kingdom; ^4^Faculty of Science, Prince of Songkla University, Songkhla, Thailand; ^5^European Bioinformatics Institute, European Molecular Biology Laboratory, Hinxton, Cambridge, United Kingdom; ^6^London School of Hygiene and Tropical Medicine, London, United Kingdom; ^7^Faculty of Allied Health Sciences, Thammasat University, Pathumtanee, Thailand; ^8^Faculty of Medicine Siriraj Hospital, Mahidol University, Bangkok, Thailand

**Keywords:** *Acinetobacter baumannii*, antibiotic resistance, phylogenomics, surface structures, complement, global clone 2

## Abstract

Antibiotic resistant strains of *Acinetobacter baumannii* are responsible for a large and increasing burden of nosocomial infections in Thailand and other countries of Southeast Asia. New approaches to their control and treatment are urgently needed and an attractive strategy is to remove the bacterial polysaccharide capsule, and thus the protection from the host’s immune system. To examine phylogenetic relationships, distribution of capsule chemotypes, acquired antibiotic resistance determinants, susceptibility to complement and other traits associated with systemic infection, we sequenced 191 isolates from three tertiary referral hospitals in Thailand and used phenotypic assays to characterize key aspects of infectivity. Several distinct lineages were circulating in three hospitals and the majority belonged to global clonal group 2 (GC2). Very high levels of resistance to carbapenems and other front-line antibiotics were found, as were a number of widespread plasmid replicons. A high diversity of capsule genotypes was encountered, with only three of these (KL6, KL10, and KL47) showing more than 10% frequency. Almost 90% of GC2 isolates belonged to the most common capsule genotypes and were fully resistant to the bactericidal action of human serum complement, most likely protected by their polysaccharide capsule, which represents a key determinant of virulence for systemic infection. Our study further highlights the importance to develop therapeutic strategies to remove the polysaccharide capsule from extensively drug-resistant *A. baumanii* during the course of systemic infection.

## Introduction

*A. baumannii* is an opportunistic pathogen that can cause potentially lethal nosocomial infections (Howard et al., 2012). These are frequently a result of trauma, surgery, catheterization or endotracheal intubation (Chopra et al., 2014), and *A. baumannii* can escape the local immune reaction by evading neutrophils, macrophages and complement (C') (Russo et al., 2008; García-Patiño et al., 2017). This immune escape therefore necessitates the use of antimicrobials, and the key determinant of clinical outcome of *A. baumannii* infection is treatment failure due to the high number of antibiotic resistant strains (Wong et al., 2017).

Multidrug resistant (MDR) strains of *A. baumannii* have spread rapidly over recent decades (Zarrilli et al., 2013; Hamidian and Nigro, 2019). The high prevalence of strains resistant to nearly all antibiotics, especially well-tolerated cephalosporins and carbapenems, has led to the revival of drugs considered to be of last resort such as polymyxins (Falagas and Kasiakou, 2005; Sahbudak Bal et al., 2018) for systemic administration. However, resistance to colistin is now more prevalent and polymyxins are now used less widely due to serious side effects associated with these agents (Sahbudak Bal et al., 2018). As a consequence, the World Health Organization has identified carbapenem-resistant *A. baumannii* (CRAB) as the greatest bacterial threat to global human health and the top priority pathogen for development of new antibiotics (Tacconelli et al., 2018).

Recent surveillance data indicates that *A. baumannii* causes under 2% of healthcare associated infections in the United States (Sievert et al., 2013; Bulens et al., 2018) but prevalence is much higher in Southern and South Eastern Asia, where it is frequently the major nosocomial infectious agent (Suwantarat and Carroll, 2016). The burden of *A. baumannii* infection is particularly severe in Thailand, with isolates accounting for 15–16% of hospital-acquired bacteremia cases and displaying very high levels (70–88%) of carbapenem resistance, and mortality rates in excess of 60% due to MDR *A. baumannii* bacteremia (Chaisathaphol and Chayakulkeeree, 2014; Hongsuwan et al., 2014; Suwantarat and Carroll, 2016; Hsu et al., 2017; Sirijatuphat et al., 2018). Presence of the over-expressed carbapenemase *bla*_OXA–__23_, or *bla*_OXA–__51_ in combination with IS elements, account for most of the CRAB phenotypes (Figueiredo et al., 2009; Teo et al., 2015; Wong et al., 2017). Molecular typing identified three European clones; two have spread globally and are now identified as GC1 and GC2 (Higgins et al., 2010; Hamidian and Nigro, 2019) and the majority of isolates from Asia belong to global clone 2 (GC2) (Kim et al., 2013; Kamolvit et al., 2015).

The large majority of *A. baumannii* strains produce a substantial capsular polysaccharide that protects them from external threats (Kenyon and Hall, 2013), and an attractive treatment option is enzymatic removal of the protective capsules (Mushtaq et al., 2004; Lin et al., 2014; Negus et al., 2015); capsule-free mutants were highly susceptible to C'-mediated attack (Lees-Miller et al., 2013), in marked contrast to their encapsulated parent strains. A major advantage of this approach is that it circumvents the accumulation of antibiotic resistance determinants, but has the potential disadvantage that variation of the capsular polysaccharide may limit the utility of individual depolymerases as found in bacteriophages or other organisms, which typically hydrolyze only one or a limited number of capsular types (Oliveira et al., 2017; Hernandez-Morales et al., 2018; Lin et al., 2018; Singh et al., 2018).

We report a detailed characterization of 191 recent isolates from three major hospitals in Thailand using whole-genome sequencing and functional assays, with particular reference to their surface properties and antibiotic resistance profiles. We also sought to identify factors that contribute to the capacity of GC2 isolates to cause infection through increased virulence (Zarrilli et al., 2013), using genomic data and bioassays, in relation to the role of the capsule in the determination of resistance to C'-mediated attack.

## Materials and Methods

### Bacterial Isolates

**FIGURE 1:**
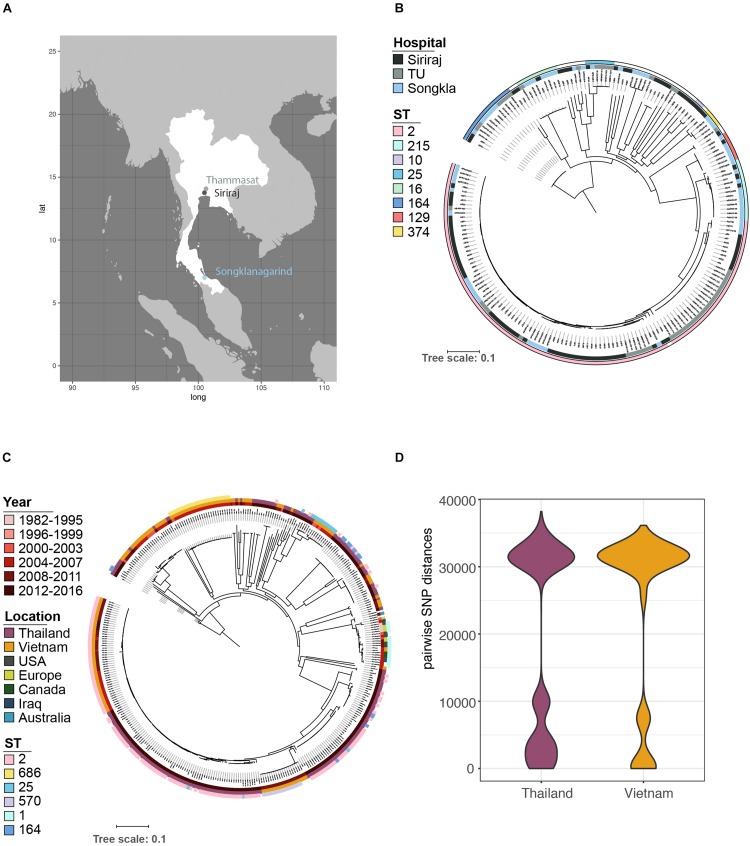
The population structure of *A. baumannii* isolated from a variety of infections in April 2016 at three major Thai hospitals. **(A)** Samples were obtained from geographically distinct regions of the country. **(B)** Core gene phylogeny showed that the bacterial populations were circulating amongst the three hospitals; no single lineage dominated at any one location. **(C)** Our data in context with the global population structure based on published data. **(D)** A more detailed comparison of the data structure of pairwise SNP distances shows a similar distribution between our samples and a recent study from one hospital in Vietnam (Schultz et al., 2016), with a similarly high prevalence of ST2 **(C)**, but also a considerable number of more distantly related isolates from other regions.

A total of 191 *A. baumannii* isolates were cultured from wound pus, sputum, urine, blood, and excised tissue at the clinical microbiology laboratories of three tertiary referral hospitals in Thailand (Figure 1A). Bacteria were initially identified by routine biochemical tests implemented for identification of Gram-negative bacteria. Species were further confirmed by whole-genome sequencing and sequence typing as below. The hospitals were Thammasat University Hospital, Pathum Thani Province (47 isolates; April 2016), Siriraj Hospital, Bangkok (84 consecutive isolates; April 2016) and Songklanagarind Hospital, Hat Yai, Songkhla Province (60 isolates; August 2016). Siriraj is the largest hospital in Thailand with 2,300 beds, 1,000,000 outpatients per annum and 80,000 inpatients per annum; equivalent figures for Songklanagarind are 846, 1,019,375, and 40,936 and for Thammasat 601, 384,088, and 40,745 (data from 2017). Details of these isolates are given in Supplementary Table S1. Susceptibilities to clinically relevant antibiotics were determined using the Vitek 2 system (Bosshard et al., 2006).

### Genome Sequencing, Assembly, and Annotation

Genomic DNA was extracted and sequenced using Illumina-B HiSeq X paired-end sequencing. Annotated assemblies were produced according to (Page et al., 2016a). Sequence reads were assembled *de novo* with Velvet v1.2 (Zerbino and Birney, 2008) and VelvetOptimiser v2.2.5 (Gladman and Seemann, 2008). Reads were annotated using PROKKA v1.11 (Seemann, 2014). The stand-alone scaffolder SSPACE (Boetzer et al., 2011) was used to refine contig assembly; sequence gaps were filled using GapFiller (Boetzer and Pirovano, 2012). Genomes with greater than 5% contamination levels as determined by Kraken (Wood and Salzberg, 2014), fully assembled genomes of less than 4.5 Mpb or comprising 500 or more contigs were removed. Putative genomes with less than 60% sequence similarity with the reference genome were assessed with CheckM (Parks et al., 2015) for genome completeness and contamination; isolates with greater than 3% contamination levels were excluded from the study. SNPs were called against the *A. baumannii* reference genome to identify heterozygous SNPs, and isolates with more than 2% were removed from further analysis (Page et al., 2016a), resulting in the 191 genomes analyzed in this study. As we could also observe several *gdhB* duplicate sequences, a known problem of the Oxford MLST scheme (Bartual et al., 2005; Gaiarsa et al., 2019), sequence types were assigned and are reported only based on the Pasteur scheme (Diancourt et al., 2010; Page et al., 2016). Novel sequence types were assigned for non-typeable isolates through the PubMLST (Supplementary Table S1), three isolates could not be assigned as the assemblies were missing one allele.

### Phylogenetic Analyses

The pan genome for the global and Thai isolate analyses was determined with Roary (Page et al., 2015) using a Protein BLAST identity of 95% and a core definition of 99%. SNPs were extracted from the core gene alignment using SNP sites (Page et al., 2016b) and the output used to run RAxML v8.2.8 (Stamatakis, 2014) to calculate the phylogenetic tree with 100 bootstraps under the GTR time-reversible model. The resulting alignment for the global dataset was also used to determine pairwise SNP distances with the dist.gene function from the ape package in R (Paradis et al., 2004). To place our isolates in a broader context, we compared them with recently published sequence data of *A. baumannii* causing ventilator-associated pneumonia in the intensive care unit of a Vietnamese hospital, in addition to data from several other published studies (Supplementary Table S2).

### Antibiotic Resistance and Traits Associated With Infection

Antibiotic resistance genes were detected with the curated version of the ARG-ANNOT database available at the SRST2 site (Gupta et al., 2014; Inouye et al., 2014), *rpoB* SNP mutations were assessed comparing the sequences against described resistance mutations (Giannouli et al., 2012; Pérez-Varela et al., 2017), and virulence factors with VFDB (Chen et al., 2016), using the read-based search program ARIBA (Hunt et al., 2017). Plasmid replicons were detected with a custom database composed of 30 genes involved in plasmid replication, stabilization and mobilization from *Acinetobacter* plasmids (Bertini et al., 2010; Salto et al., 2018); some additional plasmids (Gao et al., 2011; Hamidian et al., 2012, 2016; Zhang et al., 2013; Jones et al., 2014; Blackwell and Hall, 2017; Hamidian et al., 2017) were also included (full database Supplementary Dataset S1); and analyses were undertaken using ARIBA software v2.12.1 (Hunt et al., 2017). To account for potential variation in surface proteins or other virulence factors, a custom-made collection of *A. baumannii* virulence factors (Supplementary Table S6) was searched against our isolates using phmmer (Eddy, 2011; Eijkelkamp et al., 2011, 2014; Harding et al., 2013; Scott et al., 2014; Weber et al., 2015; Lee et al., 2017). Representations of trees and metadata were performed using iTOL (Letunic and Bork, 2016) and the ggplot2 and ggtree packages in R (Wickham, 2009; Yu et al., 2018). KL and OCL genotypes of our isolates were identified using the capsule identification program kaptive, based on a curated *A. baumannii* specific database (Wyres et al., 2019; Supplementary Table S1).

### C' Susceptibility

Commercial (MP Biomedicals, United Kingdom) pooled human serum was stored and used to determine susceptibility to C', essentially as previously described (Loraine et al., 2018). Early mid-logarithmic-phase Luria-Bertani (LB) broth cultures of *A. baumannii* were washed three times with 200 μl of gelatin-veronal-buffered saline containing Mg^2+^ and Ca^2+^ (GVB^++^; pH 7.35) and suspended in 400 μl of GVB^++^. The suspensions (200?μl) were mixed with 390 μl of pre-warmed (37°C) normal human serum to give a final concentration of ∼1 × 10^6^ CFU, the mixtures incubated at 37°C for 3 h and bacteria quantified by serial dilution and overnight incubation on LB agar (see Supplementary Table S3 for all raw data). The 45 GC2 isolates were exposed to 66% normal human serum and enumerated bacterial survivors over a 3 h incubation period (Malke, 1986). Isolates were assigned to one of three categories: resistant (R), showing no (or only transient) reduction in viable count during the incubation period; delayed susceptible (DS), displaying significant (∼90%) survival after 1 h and low survival (<10%) after 3 h incubation; the inocula of rapidly susceptible (S) isolates were reduced to below 10% after 1 h incubation. All experiments were performed in duplicate and results expressed as percent survival over this time period. Pre-warmed, heat-inactivated human serum (56°C, 30 min) served as control. All raw data is given in Supplementary Table S3.

### Capsule Measurements

The size of the capsule for each isolate was determined by negative staining with India ink, microscopic imaging and calculation of the area occupied by the capsule using CellProfiler image analysis software (v3.1.9; Lamprecht et al., 2007). One bacterial colony was resuspended in PBS and mixed in a 1:1 ratio with India Ink stain (BD India Ink Reagent Dropper) and applied to a microscope slide with a coverslip. Microscopic imaging with a Zeiss Axiostar plus transmitted light microscope fitted with an Olympus SC30 digital camera and using a 100× oil immersion lens and embedded scale bar. All raw data is given in Supplementary Table S4.

### Motility

Swarming and twitching motility were assayed by the subsurface agar method (Clemmer et al., 2011) using LB broth containing either 0.4 or 0.8% agar. Briefly, freshly grown cultures of *A. baumannii* were stabbed to enable spread of bacteria on the surface of 0.4% agar plates for swarming motility and the interphase between the bottom of the Petri dish and the 0.8% agar layer for twitching motility. The plates were incubated at 37°C for 48 h: positive swarming motility was defined as a zone greater than 10 mm around the site of inoculation. For twitching motility at the interstitial surface between the agar and the petri dish, the agar was discarded, and bacteria visualized by staining stained with 0.2% crystal violet. Positive twitchers were defined as those cultures that showed a zone diameter greater than 5 mm. Assays were performed a minimum of three times for each isolate. All raw data is provided in Supplementary Table S5.

## Results

### Major Lineages Are Circulating in the Region

Phylogenetic analysis identified several lineages circulating in all the three hospitals (Figures 1A,B). The majority of isolates belong to GC2 (*n* = 106/191), represented exclusively by sequence type 2 (ST2) of the Pasteur scheme. No isolates belonging to GC1 were identified, a key clonal group in the evolution of multi-drug resistance in *A. baumannii* (Holt et al., 2016). Non-GC2 isolates belonged to ST164 (*n* = 14; 7.3%), ST215 (*n* = 13; 6.8%), ST16 (*n* = 9; 4.7%), ST25 (*n* = 6; 3.1%), ST129 (*n* = 6; 3.1%), ST374 (*n* = 4; 2.1%), and ST10 (*n* = 2; 1.0%); three isolates could not be sequence-typed, most likely due to low-quality genomes, and thus missing one of the MLST alleles. The high prevalence of GC2 and lack of GC1 of our dataset from 2016 closely resembles the population structure from the Vietnamese hospital outbreak (Schultz et al., 2016) over the period 2009-12 (Figure 1C); both datasets include a considerable number of deep branching lineages. These similarities in population structure are also mirrored when comparing the distribution of pairwise single-nucleotide polymorphisms (SNPs) between the datasets from Vietnam and Thailand (Figure 1D).

### Antimicrobial Resistance

**FIGURE 2:**
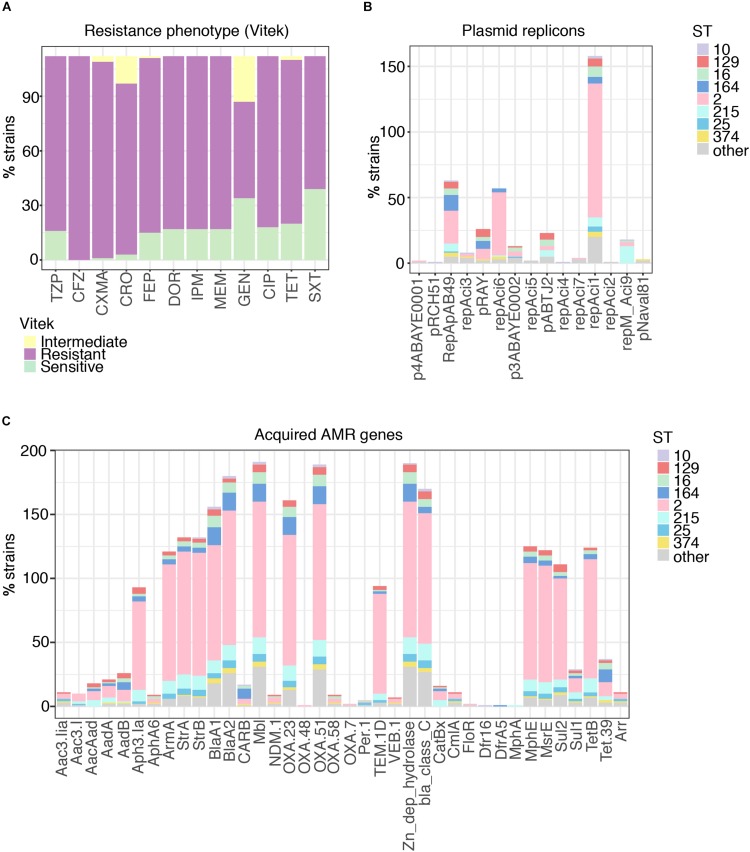
Phenotypic resistance of *A. baumannii* at high levels for all antimicrobial classes. **(A)** Resistance phenotypes measured on site at time of isolation clearly demonstrate the highly problematic levels of resistance in *A. baumannii*, with > 70% non-sensitive against all tested classes. TZP, piperacillin-tazobactam; CFZ, cefazolin; CXMA, cefuroxime axetil; CRO, ceftriaxone; FEP, cefepime; DOR, doripenem; IPM, imipenem; MEM, meropenem; GEN, gentamicin; CIP, ciprofloxacin; TET, tetracycline; SXT, trimethoprim-sulfamethoxazole. **(B)** Distribution of plasmids carried by *A. baumannii* in relation to sequence type (ST). **(C)** Distribution of acquired antimicrobial resistance genes carried by *A. baumannii* in relation to sequence type (ST).

Phenotypic resistance profiles for 115 of the strains confirmed the very high levels of antibiotic resistance encountered with clinical isolates of *A. baumannii*, especially against β-lactam agents (e.g., ceftriaxone: 115/115, 100%), including carbapenems (Figure 2A; 98/115, 85.2%), but also against other major antibiotic classes: fluoroquinolones [98/115, 85.2% resistant/intermediate (R/I)], aminoglycosides (79/115, 68.7% R/I) and trimethoprim (76/115, 66.1% R/I), and multidrug resistance was, as expected, associated with a high number of acquired resistance genes (Figures 2A,C and Supplementary Figures S1, S2) indicating either gain through larger elements carrying several genes as previously described as a key driver for *A. baumannii* resistance (Bonomo and Szabo, 2006; Post and Hall, 2009). *bla*_OXA–__23_, the most prominent carbapenem resistance gene, is present in 85.2% of imipenem resistant strains (Figure 2C). Few isolates carried the *bla*_NDM–__1_ gene and a low number of acquired *ampC* genes were detected (Figure 2C and Supplementary Figure S2). We also note the presence of the *arr* gene, as well as *rpoB* mutations, conferring rifampicin resistance, one of the last line antimicrobials used against CRAB (Thapa et al., 2009; Durante-Mangoni et al., 2014).

### Mobile Elements

All but ten isolates contained at least one of the plasmid replicons (Supplementary Dataset S1) and 121 contained two to maximal five (Figure 2B). The three plasmid replicons detected at highest frequency were RepAci1, RepAci6, and RepApAB49. Each of these plasmid types were found across a number of STs, although RepAci1 plasmids were present in almost all the ST2 isolates (102/106) and RepApAB49 was found in 12/14 ST164 isolates. Recently, a RepAci1 plasmid was shown to be mobilized by a co-residing conjugative RepAci6 plasmid (Blackwell and Hall, 2019), and these two replicons co-occur in the genomes of 56 isolates; RepAci6 only was detected in one isolate, and RepAci1 only in 46. RepAci6 plasmids were the most common self-transmissible plasmids detected. Plasmid replicons detected frequently included those matching pRAY^∗^, which is often associated with the *aadB* gene (Hamidian et al., 2012), RepAci3, p3ABAYE, pABTJ2, and RepAci9. RepMAci9 was detected in all thirteen ST215 isolates. Seven plasmid types were present in low frequency (Figure 2B) and an additional fifteen plasmid sequences were not detected in the Thai collection (Supplementary Dataset S1).

### Genes Associated With Capsules and Outer Core

**FIGURE 3:**
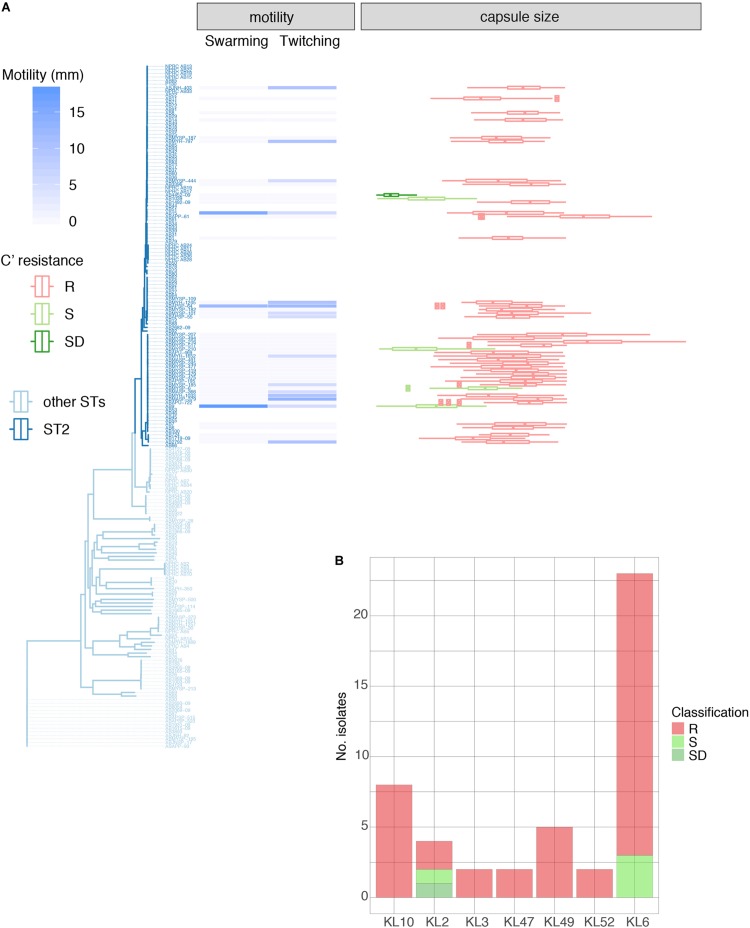
Properties of 46 GC2 *A. baumannii* Thai isolates belonging to the common capsule genotypes encountered in this study. **(A)** Motility measurements, capsule size, and C' susceptibility in phylogenetic context. **(B)** C' resistance profiles stratified by capsule type.

*A. baumannii* does not contain genes involved in lipopolysaccharide (LPS) O-antigen ligase activity (Kenyon and Hall, 2013; Weber et al., 2015), synthesizing instead a lipooligosaccharide (LOS) consisting of an outer core oligosaccharide (OCL) linked to Lipid A (Kenyon and Hall, 2013; Kenyon et al., 2014a); at least twelve distinct OCL structures have been inferred from genomic data (Kenyon et al., 2014b). We mapped all Thai isolates against an *A. baumannii* specific databases for capsular and LOS loci (KL and OCL, resp.; Supplementary Figure S3; Wyres et al., 2019). In similar fashion to the Vietnam study (Schultz et al., 2016), we noted a high diversity of KL within both GC2 and non-GC2 isolates. KL6 (15.2%), KL10 (15.7%), KL47 (11.0%), KL2 (8.4%), KL52 (7.9%), KL3 (7.3%), KL49 (6.3%), KL24 (5.8%), KL14 (3.1%), and KL28 (2.1%) were frequently encountered and KL32, KL63, KL57, KL8, KL108, KL19, KL113, KL116, KL60, KL43, KL37, KL9, KL125, and KL7 were represented in 2% or fewer isolates. KL could not be determined in 15 isolates (7.9%). KL2 and KL49 were found at least twice in the Vietnam isolates although we did not detect KL58, strongly represented in Schultz et al. (2016). Eight distinct capsule loci in our GC2 isolates were detected in isolates from all three hospitals during April 2016 and provide a challenge for novel therapies targeting bacterial cell surfaces. Furthermore, seven distinct LOS loci were detected amongst the Thai isolates (Supplementary Figure S3 and Supplementary Table S1). The majority of GC2 isolates carried genes for OCL1 biosynthesis (91 isolates, 85.8% of all GC2 isolates, 61.8% total), whilst the other types, OCL2 (6.8%), OCL3 (4.2%), OCL4 (1.6%), OCL5 (15.7%), OCL6 (4.7%), and OCL7 (5.2%) were also widely distributed amongst our isolates; there was, however, no clear association between K- and LOS-types (Figure 3).

### Linking Virulence-Associated Phenotype, Site of Isolation, and Genotype in GC2

We examined 45 GC2 isolates belonging to the major capsule types identified in the Thai collection: KL10 (eight isolates), KL2 (4), KL3 (2), KL47 (2), KL49 (5), KL52 (2), and KL6 (22). Although *A. baumannii* strains lack flagella, the species displays type IV-mediated twitching motility that facilitates spreading on abiotic surfaces (Vijayakumar et al., 2016), and it has been linked to the capacity of strains to cause systemic infection (Harding et al., 2018). Only six of our 45 GC2 isolates were derived from blood samples but all displayed twitching motility (Table 1, Supplementary Table S5, and Figure 3A). In contrast, none of six tissue isolates and only a minority of sputum isolates (10/33) were motile in this fashion. The capacity to swarm on semi-solid agar (surface-associated motility; Harding et al., 2018) can also be linked to a more virulent phenotype (Eijkelkamp et al., 2011; Tipton and Rather, 2017). 24/45 of the Thai GC2 isolates displayed surface-associated (swarming) motility; 6/6 of these were from tissue samples and 18/33 from sputum (Table 1 and Figure 3A). Three isolates from sputum exhibited both forms of motility.

**TABLE 1:** Properties of GC2 *A. baumannii* clinical isolates.

Thai strain ID	Hospital	KL	OCL	ST	Sample source	Motility (mm)		C' Susceptibility^b^
						
						Swarming^c^	Twitching^d^	
ABMYSP-109	Thamm^*a*^	KL10	OCL1	2	Sputum	≤10	15	R
ABMYH-1245	Thamm	KL10	OCL1	2	Blood	≤10	10	R
ABAPSP-55	Thamm	KL10	OCL1	2	Sputum	≤10	5	R
ABAPSP-64	Thamm	KL10	OCL1	2	Sputum	12	12	R
ABMYSP-101	Thamm	KL10	OCL1	2	Sputum	≤10	5	R
ABMYSP-182	Thamm	KL10	OCL1	2	Sputum	14	<5	R
ABMYSP-187	Thamm	KL10	OCL1	2	Sputum	14	<5	R
ABMYH-797	Thamm	KL10	OCL1	2	Blood	≤10	10	R
AB1039	Songkla	KL2	OCL1	2	Sputum	19	<5	S
AB1492-09	Songkla	KL2	OCL1	2	Sputum	16	<5	R
AB3396	Songkla	KL2	OCL1	2	Tissue	15	<5	R
AB4452-09	Songkla	KL2	OCL1	2	Sputum	≤10	<5	DS
AB11	Siriraj	KL3	OCL1	2	Sputum	≤10	<5	R
ABJNH-403	Thamm	KL3	OCL1	2	Blood	≤10	10	R
AB15	Siriraj	KL47	OCL1	2	Sputum	15	5	R
ABAPP-61	Thamm	KL47	OCL1	2	Tissue	15	<5	R
AB8	Siriraj	KL49	OCL1	2	Sputum	15	<5	R
AB14	Siriraj	KL49	OCL1	2	Sputum	17	<5	R
AB724	Songkla	KL49	OCL1	2	Sputum	14	<5	R
AB1719-09	Songkla	KL49	OCL1	2	Tissue	15	<5	R
AB2792	Songkla	KL49	OCL1	2	Blood	≤10	10	R
AB1	Siriraj	KL52	OCL1	2	Tissue	14	<5	R
ABMYSP-444	Thamm	KL52	OCL1	2	Sputum	≤10	5	R
AB6	Siriraj	KL6	OCL1	2	Sputum	18	<5	R
AB7	Siriraj	KL6	OCL1	2	Sputum	17	<5	R
AB9	Siriraj	KL6	OCL1	2	Sputum	18	5	S
ABMYSP-185	Thamm	KL6	OCL1	2	Sputum	≤10	5	R
ABMYSP-216	Thamm	KL6	OCL1	2	Sputum	16	<5	R
ABMYH-1652	Thamm	KL6	OCL1	2	Blood	≤10	5	R
ABMYSP-475	Thamm	KL6	OCL1	2	Sputum	18	<5	R
ABMYSP-477	Thamm	KL6	OCL1	2	Sputum	15	<5	R
ABMYSP-479	Thamm	KL6	OCL1	2	Sputum	15	<5	R
ABMYSP-517	Thamm	KL6	OCL1	2	Sputum	≤10	<5	R
ABMASP-366	Thamm	KL6	OCL1	2	Sputum	≤10	5	R
ABMASP-379	Thamm	KL6	OCL1	2	Sputum	≤10	13	R
ABMASP-491	Thamm	KL6	OCL1	2	Sputum	≤10	<5	R
ABAPSP-195	Thamm	KL6	OCL1	2	Sputum	≤10	<5	R
ABAPU-469	Thamm	KL6	OCL1	2	Tissue	11	<5	R
ABAPU-722	Thamm	KL6	OCL1	2	Tissue	16	<5	R
ABMYSP-494	Thamm	KL6	OCL1	2	Sputum	16	<5	R
ABMYSP-6	Thamm	KL6	OCL1	2	Sputum	16	<5	S
ABMYSP-207	Thamm	KL6	OCL1	2	Sputum	≤10	<5	R
ABMYSP-210	Thamm	KL6	OCL1	2	Sputum	≤10	<5	S
ABMYSP-245	Thamm	KL6	OCL1	2	Sputum	≤10	<5	R
ABMYH-1033	Thamm	KL6	OCL1	2	Blood	≤10	10	R

*^a^Thamm: Thammasat Hospital; Songkla: Songklaragarind Hospital; Siriraj: Siriraj Hospital. ^*b*^Complement reactivity: R, Resistant; DS, Delayed susceptible; S, Rapidly susceptible. ^*c*^Values less than 10 mm are considered negative (Vijayakumar et al., 2016). ^*d*^Values less than 5 mm are considered negative (Vijayakumar et al., 2016).*

Many loci that have been linked to the capacity of *A. baumannii* to colonize, invade and disseminate within the host, such those encoding adhesins, capsules, quorum sensors, iron sequestering systems and other nutrient scavengers (Harding et al., 2018), are essential or advantageous for survival in its natural habitat, predominantly soil and water (Baumann et al., 1968). The distribution of genes based on a publicly available virulence factor database is shown in Supplementary Figure S4, but whilst there are clear differences, no trend (for example increased prevalence in GC2) could be observed. As expected, siderophores, adhesins involved in biofilm formation and maintenance, and a variety of genes determining capsule biosynthesis are widely distributed among the isolates.

### GC2 Capsule Size Correlates With Survival in Human Serum

A large proportion (40/45, 88.9%) were refractory to C'-mediated killing; of the remainder, only four were categorized as S (Table 1). All KL10, KL3, KL47, KL49, and KL52 isolates belonged to the R group, with only KL2 (2/4) and KL6 (3/23) capsule types displaying any degree of C' susceptibility (Figures 3A,B). All 45 GC2 isolates examined were encapsulated. The C' susceptible isolates elaborated significantly smaller capsules than R *A. baumannii* (R, mean 1.62 mm^2^; DS, 0.31 mm^2^; S, 0.81 mm^2^); all capsule locus predictions however showed a perfect or almost perfect match, emphasizing that the capsule biosynthesis locus is likely intact (Figure 3A). Capsules containing sialic acids protect Gram-negative bacteria from C' attack (Rautemaa and Meri, 1999), and *N*-acetylneuramininc acid and related non-ulosonic and sialic acid structures have recently been found as repeat-unit constituents or as modifications of capsule structures in hypermucoviscous *K. pneumoniae* (Lin et al., 2014) and *A. baumannii* (Vinogradov et al., 2014; Kenyon et al., 2015; Singh et al., 2018), and associated with increased infectivity. Biosynthesis of sialic acids begins with the conversion of UDP-*N*-acetylglucosamine to UDP and *N*-acetylmannosamine by the hydrolyzing 2-epimerase NeuC; a homolog of this enzyme has been described for *A. baumannii* and its crystal structure determined (Ko et al., 2018). In our set of genomes, the *neuC* homolog (A0A154EJU5_ACIBA) was found only in the genomes of the five C' resistant isolates carrying genes for biosynthesis of the K49 capsular polysaccharide and is indeed a component of the KL49 locus and should thus correctly be annotated as *lgaC*; the repeat unit of the K49 capsular polysaccharide is composed of α-L-fucosamine, α-D-glucosamine and the non-ulosonic acid α-8-epi-legionaminic acid (Vinogradov et al., 2014).

OmpA, one of most abundant porins, is also known to bind factor H in human serum (Kim et al., 2009), and implicated to prevent C' mediated killing; it is however present in all our GC2 strains (Supplementary Figure S4). A more detailed analysis of putative factors explaining the phenotypes (type IV pili, surface proteins, secretion systems, biofilm formation (Weber et al., 2015; Lee et al., 2017; Supplementary Table S6) of the 47 GC2 isolates showed no differences that correlated with any of the phenotypes tested. We also included sequence analyses of PilA, which has been shown to influence twitching motility (Ronish et al., 2019), however, the sequences from all phenotyped isolates were identical.

## Discussion

Multi-drug resistant *A. baumannii* infections are rapidly increasing and require the use of last-line treatments such as colistin. An additional challenge further narrowing the spectrum of available options for highly resistant *A. baumannii* infections is that last-line treatments available often overlap with other highly problematic infections. One example is the use of rifampicin in combination with colistin against CRAB, which is also one of the last options to treat the increasing number of multi-drug resistant tuberculosis (MDR TB) cases, and use of rifampicin is therefore restricted in use against organisms other than MDR TB (Thapa et al., 2009; Durante-Mangoni et al., 2014; Leite et al., 2016; Seijger et al., 2019). There is therefore a growing interest in the potential of non-antibiotic therapeutic approaches including bacteriophage-derived capsule depolymerases as treatment alternative to antimicrobial chemotherapy (Waldor et al., 2005; García-Quintanilla et al., 2013; Seijger et al., 2019).

We present the analysis of a set of 191 *A. baumannii* clinical isolates from three major hospitals in Thailand with very high levels of drug resistance. The population structure is biased toward the major clone GC2, as has been observed in other studies in geographic proximity (Schultz et al., 2016). However, the inter-mixed origins of closely related isolates from all three hospitals clearly indicates that both GC2 as well as less dominant sequence types are circulating in the region, and are frequently (re)introduced into hospitals, as opposed to a clonal outbreak within one hospital. In addition to the phylogenetic diversity (almost 50% non-GC2 isolates) and the even spread across the three hospitals, we show that there is a high degree of strain-to-strain capsule variability, and development of depolymerase therapeutics will need to account for the challenge of a wide range of capsule types. Nevertheless, a recent study has demonstrated the potential of capsule depolymerase against *A. baumannii* in a *Galleria mellonella* (wax moth) larvae infection model and protection of both normal and immunocompromised mice from lethal peritoneal sepsis (Liu et al., 2019a).

The enzyme also sensitized the C'-resistant isolate to serum (Liu et al., 2019b), which is highly relevant as the large majority of our GC2 isolates (40/45) were C' resistant, in similar proportion to other recent studies (Sanchez-Larrayoz et al., 2017; Skerniškytë et al., 2019). LPS O-side chains prevent assembly of the C5b-9 complex by steric hindrance; *A. baumannii* however does not decorate its LOS with O-side chains but is able to modify the lipid A moiety of LOS by acylation, resulting in increased survival in blood (Bartholomew et al., 2019), which could prevent C5b-9 intercalation into the bilayer. Alternatively, there is some evidence that *A. baumannii* may prevent C' activation: resistant clinical isolates bound fH, a key inhibitor of the alternative C' pathway (Kim et al., 2009), preventing C5b-9 generation. King et al. (2009) found that clinical isolates did not bind fH but circumvented C3b deposition, again preventing C5b-9-mediated bacterial killing. Cell surface-located sialic acids are potent recruiters of fH and we therefore examined Thai GC2 isolates for evidence of *neuC*-dependent sialyl biosynthesis. The *neuC* homolog is part of the KL49 locus, however, non-ulosonic acid sugars are also found in the K2 and K6 types (Kenyon and Hall, 2013), which have C' sensitive as well as resistant phenotypes.

Current evidence indicates that C' killing of susceptible *A. baumannii* proceeds predominantly through the activation of the alternative pathway (Kim et al., 2009; Jacobs et al., 2010; Sanchez-Larrayoz et al., 2017). The lack of classical pathway killing may be due to the absence of C'-activating IgG or IgM directed against *A. baumannii* surface structures in normal human serum, suggesting that the predominant means to avoid bactericidal effects is prevention or subversion of activation of the alternative pathway. It is likely that the polysaccharide capsule is the predominant macromolecule facilitating C' resistance (Harding et al., 2018) and the four fully C' susceptible isolates in the current study elaborated less capsule than the resistant group. Capsule depolymerases as an alternative means of resolving *A. baumannii* systemic infections would thus be worth exploring but may be limited by the wide diversity of capsule types likely to be encountered in current clinical isolates.

Whilst the current focus is placed on GC2, it is important to point out that GC1 and GC2 seem to follow different strategies for interacting with the immune system and hospital environment. Whilst we report low motility and high C' resistance for GC2 and the associated genetic background, GC1 seems to follow a very different route, with high motility profiles and different adherence profiles than GC2 (Skerniškytë et al., 2019). It is thus crucial to increase active surveillance of *A. baumannii* epidemiology, as different high-risk lineages may need different approaches to reduce their burden in the clinic.

## Data Availability Statement

The datasets generated for this study can be found in the European Nucleotide Archive ERS1930151–ERS1930323.

## Author Contributions

PT, NT, and RAS conceived the study. JL, EH, GB, and PT designed experimental procedures. JL, EH, RS, and GB performed the experiments, analyzed and curated the data. SV, PS, and PK assembled the bacterial collection. PT and EH wrote the manuscript.

## Conflict of Interest

The authors declare that the research was conducted in the absence of any commercial or financial relationships that could be construed as a potential conflict of interest.

## Supplementary Material

FIGURE S1 Resistance genes and phenotypic resistance. The strains were grouped according to the number of agents in the Vitek screen the respective strains were resistant to, along the x-axis. The y-axis shows the number of strains in the relevant class, the color of the bars shows resistance (dark purple), intermediate (yellow), or sensitive (green) against the respective antimicrobial of the subplot. This shows that almost all strains are resistant against 12 reagents, sensitivity of the highly-resistant ones on the far end of the x-scale is only occasionally in sulfonamides or tetracycline, but all are fully resistant against the β-lactam class.

FIGURE S2 Presence of genes encoding antibiotic resistance in Thai *A. baumannii* isolates. The guidance tree is shown in Figure 3A. Bla, β-lactamases; AGly, aminoglycosides; MLS, macrolides; Phe, chloramphenicol; Rif, rifampin; Sul, sulfonamides; Tet, tetracycline; Tmt, trimethoprim. AMR genes were sourced from the curated version of the ARG-ANNOT database available at the SRST2 site. Isolates from Thammasat University Hospital, Siriraj Hospital, and Songklanagarind Hospital are designated TU, Siriraj, and Songkla, respectively. Sequence types are shown, as indicated in the legend. Chromosomal mutations for RpoB are also shown, we could detect potential resistance-conferring changes (Giannouli et al., 2012; Pérez-Varela et al., 2017) D525N, H535Q, and S540F.

FIGURE S3 Cell surface polysaccharide diversity of *A. baumannii* Thai isolates. Capsular (KL) and outer core loci (OCL) *in silico* typing of Thai isolates. *A. baumannii* shows considerable variation in K-type and a more conserved distribution of OCL-types.

FIGURE S4 Virulence genes associated with Thai *A. baumannii* isolates. The guidance tree is shown in Figure 3A. Antibiotic resistance genes were detected with the curated version of the ARG-ANNOT database available at the SRST2 site using ARIBA. Isolates from Thammasat University Hospital, Siriraj Hospital, and Songklanagarind Hospital are designated TU, Siriraj, and Songkla, respectively. Sequence types are shown, as indicated in the legend.

TABLES S1–S6 Properties of *A. baumannii* isolates.

DATASET S1 The custom-made plasmid replicon collection used to assign plasmid types to *A. baumannii*.
