# Supplementary figures and images for: Genomic and Phenotypic Analyses of Acinetobacter baumannii Isolates From Three Tertiary Care Hospitals in Thailand
Source: Front Microbiol. 2020 Apr 6;11:548. doi: 10.3389/fmicb.2020.00548 (PMC7153491; doi:10.3389/fmicb.2020.00548)

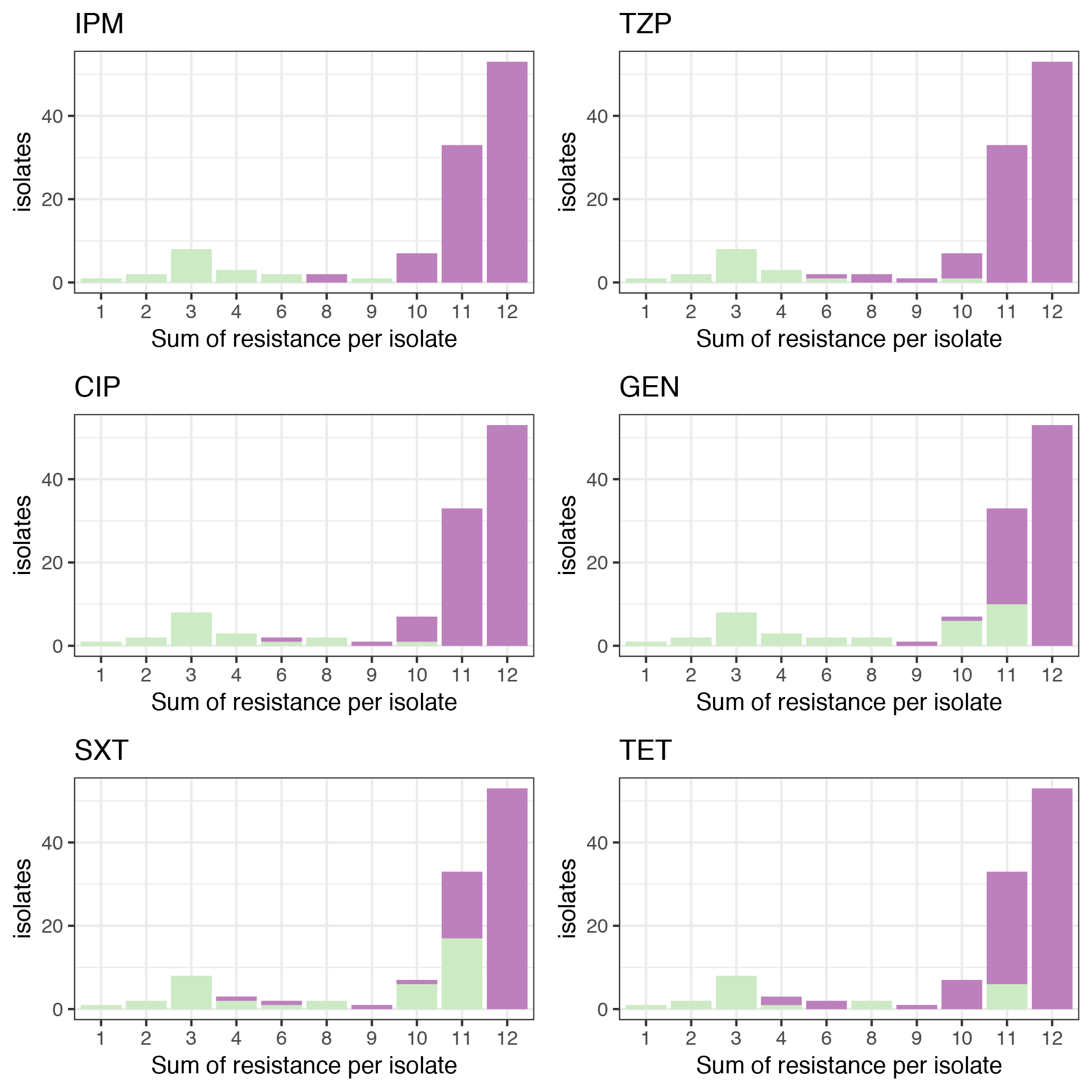

Supplement: FIGURE S1 — Resistance genes and phenotypic resistance. The strains were grouped according to the number of agents in the Vitek screen the respective strains were resistant to, along the x-axis. The y-axis shows the number of strains in the relevant class, the color of the bars shows resistance (dark purple), intermediate (yellow), or sensitive (green) against the respective antimicrobial of the subplot. This shows that almost all strains are resistant against 12 reagents, sensitivity of the highly-resistant ones on the far end of the x-scale is only occasionally in sulfonamides or tetracycline, but all are fully resistant against the β-lactam class. [file Image_1.JPEG]

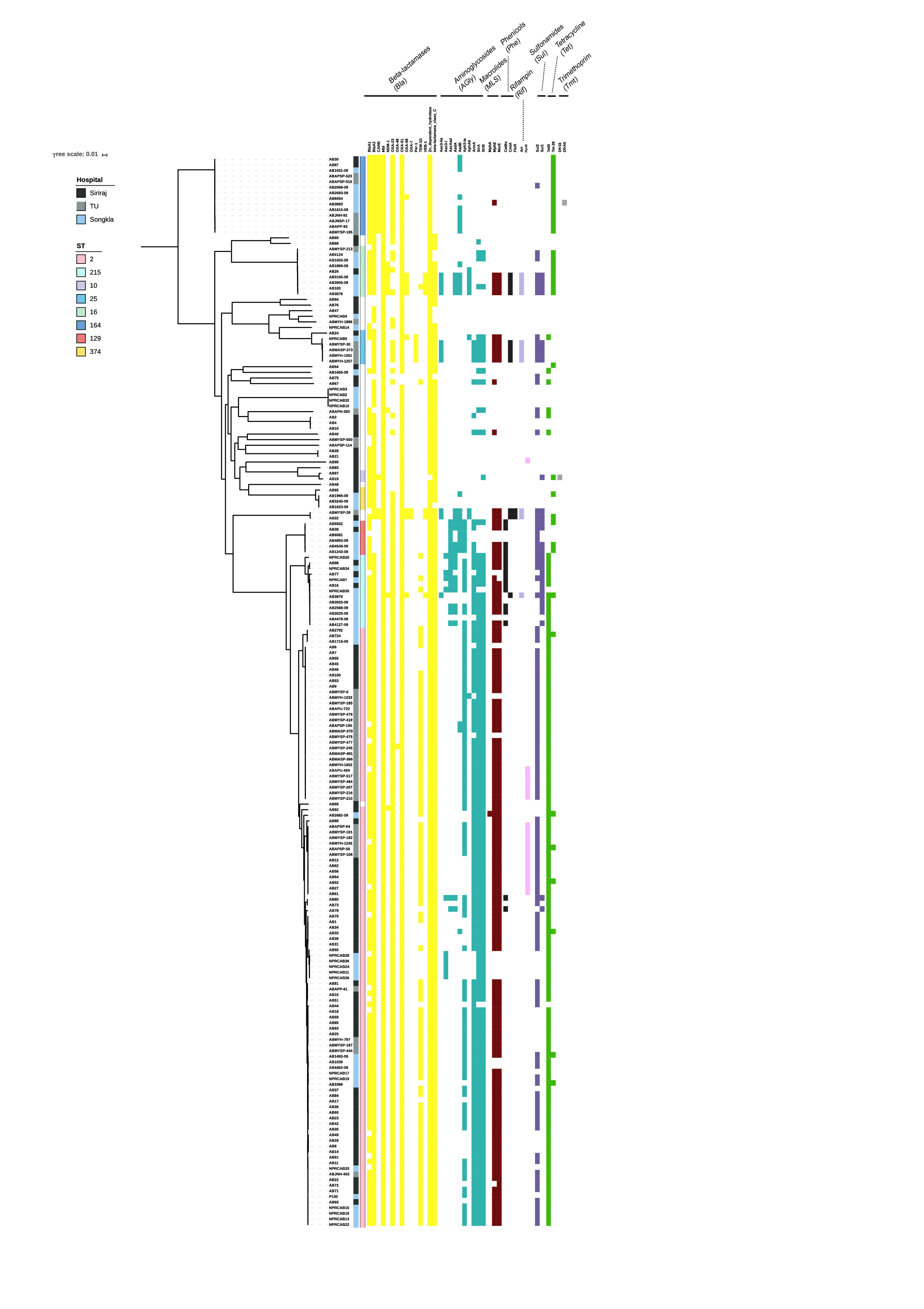

Supplement: FIGURE S2 — Presence of genes encoding antibiotic resistance in Thai A. baumannii isolates. The guidance tree is shown in Figure 3A. Bla, β-lactamases; AGly, aminoglycosides; MLS, macrolides; Phe, chloramphenicol; Rif, rifampin; Sul, sulfonamides; Tet, tetracycline; Tmt, trimethoprim. AMR genes were sourced from the curated version of the ARG-ANNOT database available at the SRST2 site. Isolates from Thammasat University Hospital, Siriraj Hospital, and Songklanagarind Hospital are designated TU, Siriraj, and Songkla, respectively. Sequence types are shown, as indicated in the legend. Chromosomal mutations for RpoB are also shown, we could detect potential resistance-conferring changes (Giannouli et al., 2012; Pérez-Varela et al., 2017) D525N, H535Q, and S540F. [file Image_2.JPEG]

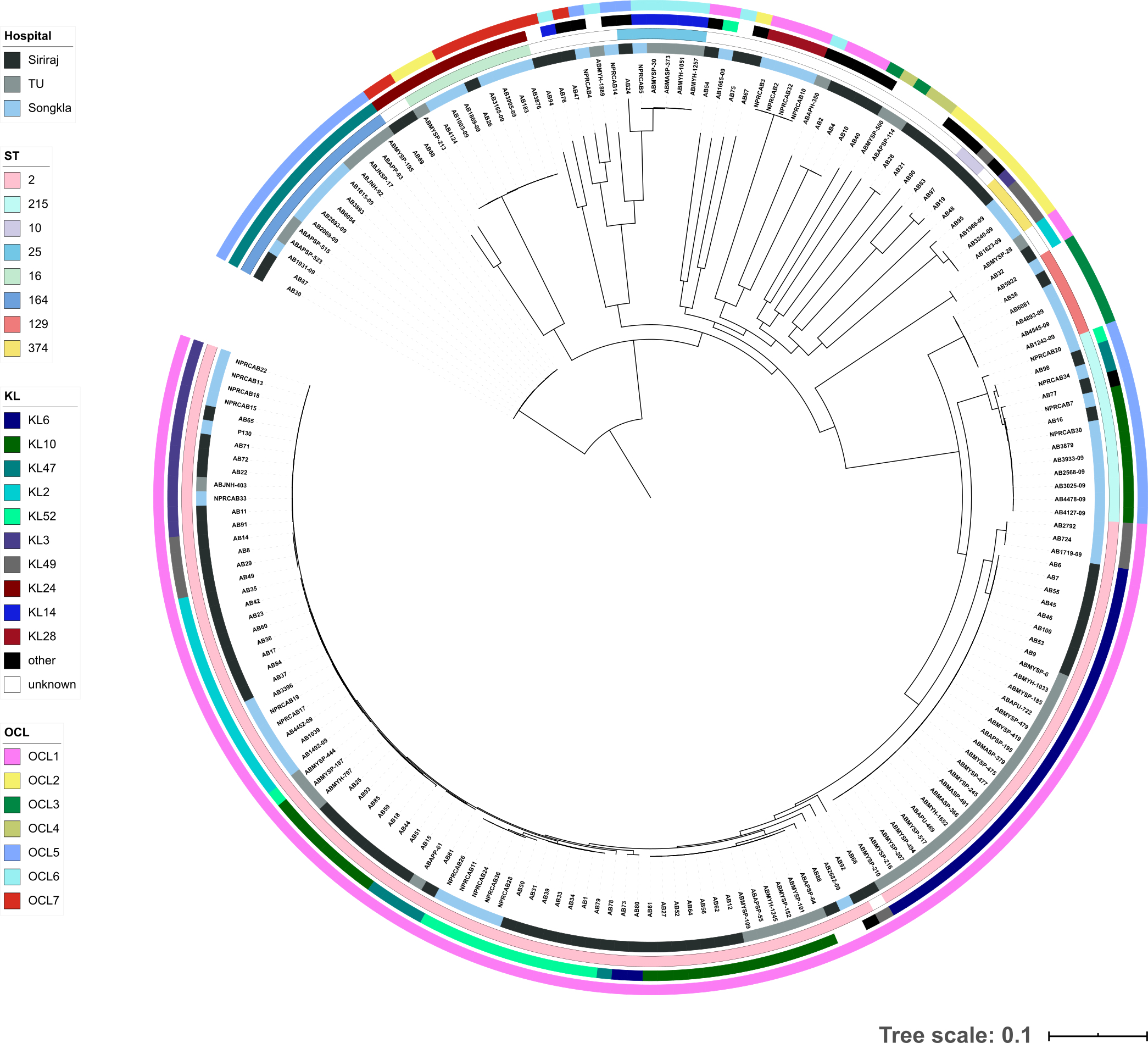

Supplement: FIGURE S3 — Cell surface polysaccharide diversity of A. baumannii Thai isolates. Capsular (KL) and outer core loci (OCL) in silico typing of Thai isolates. A. baumannii shows considerable variation in K-type and a more conserved distribution of OCL-types. [file Image_3.JPEG]

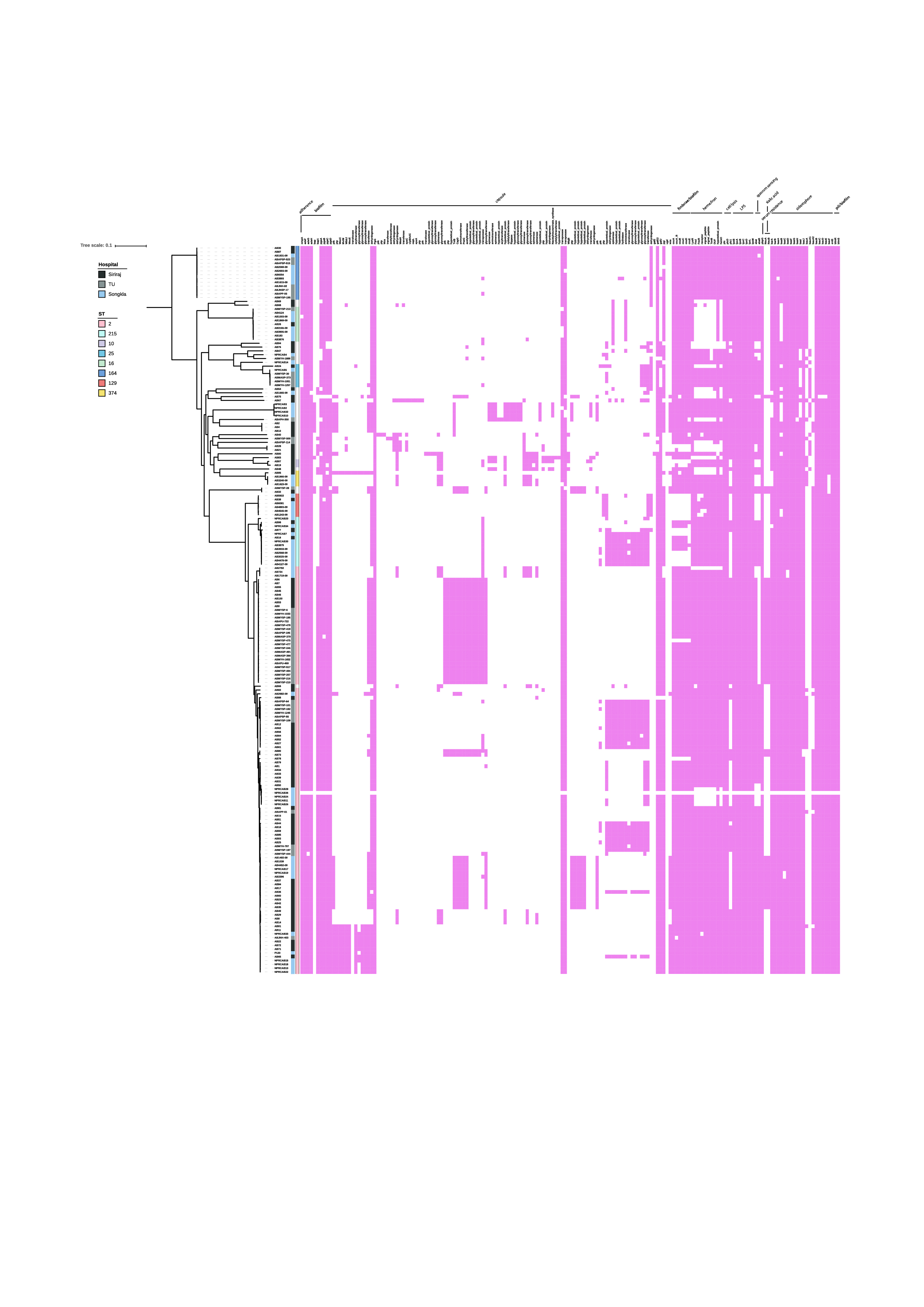

Supplement: FIGURE S4 — Virulence genes associated with Thai A. baumannii isolates. The guidance tree is shown in Figure 3A. Antibiotic resistance genes were detected with the curated version of the ARG-ANNOT database available at the SRST2 site using ARIBA. Isolates from Thammasat University Hospital, Siriraj Hospital, and Songklanagarind Hospital are designated TU, Siriraj, and Songkla, respectively. Sequence types are shown, as indicated in the legend. [file Image_4.JPEG]
