# Supplementary material for: Genomic and Phenotypic Analyses of Acinetobacter baumannii Isolates From Three Tertiary Care Hospitals in Thailand
Source: Front Microbiol. 2020 Apr 6;11:548. doi: 10.3389/fmicb.2020.00548 (PMC7153491; doi:10.3389/fmicb.2020.00548)
Supplement: DATASET S1 — The custom-made plasmid replicon collection used to assign plasmid types to A. baumannii. [file Data_Sheet_1.PDF]

> repAci1_pAbG7-1_KJ586856
atgagagatttagttgtaaaggacaatgccttaatcaacgcaagctataacttagacttagtagaaca
acgtttaattttattggctattgttgaagcaagggaaagtgggaaagggattaatgcaaatgatccat
tagaagttcatgcagatagttatatcaatcaatttggtgtacaccgtaatacagcttatcaagcctta
aaagatgcttgtaaggatttattcgcgcgtcaatttagctatcaagagaaaaaagctaatgggaatat
ccgaaatgttatgagtcgttgggtatctcaaattgcttataacgacaatgaagcaactgtagacttaa
tatttgcacctgctgttgttcccttcataacccgactggaagaacaatttactaaatatgaattacag
caagttagtagtcttagtagcgcttatgccattcgcttatatgagcttctaattcagtggcgaagcac
tggtaaaaccccaaccatagaactacaagaatttagaaagaagttaggcgttcttgataatgaatatt
tacggatggctcatttaaaagagcgtgttttagagctttcaattaaacaaataaatgagcatacggat
ataactgtaaaatatgaacagcataaaagaggacgttctatttcaggattttcttttacctttaaaca
gaagaagaaggatagcccatcaatagaaagagatccgaacactttggagctttttttcaaagatgaccg
atgctcaacggcatatgtttgcaaataaactttcagaactccctgaaatgggtcgctattcacaagga
acagaaagctaccaacagtttgctgtacgtattgctgagatgctacaagatcccgctcaattcaaaga
gctatacccatacctaaaaaaagtgggatacatgccatcaaataaaaaggacaccgtaaatggctaa

> repAci2_pABVA01_FM210331
atgagagaattagttgtaaaagacaatgccttaattaatgcaagctataacttagatttagtagaaca
acgtttaattttattggccattgttgaggcaagagaaagcgggaaaggtattaatgctaatgatcccc
ttgaagtacatgcagaaggctatatcaatcaatttggcgtacatcgcaatacggcttatcaagcatta
aaagatgcctgtaatgatttatttgcaagacaatttagctatcaaaaaataaatgaacgagggaatat
tgagaactatagatcccgttgggttagtgaaattggatatgtagataatgaagcagtggttaaactta
tctttgccccagccatagttcccttaattacacgcttagaagagcatttcactaaatacgaattgcag
caagttagtaatctcagcagtgcttatgctgttcgcttatatgaattattaattgcttggagaagtac
tggctctactcctattatagaggtaagtgatttccgtcaaagaattggcgtactcgatacagagtaca
agcgtatggaacgctttaaaactagtgtacttgagcttgctattaaacaaattaacgaacatacagat
atcactgtgaagtatgagcaacacaaaagaggtcgatcaatttcaggattctcttttacttttaaaca
gaaaaagaaggacaacccaccgatagaaagagatccgaatacgttagatctctttacaaagatgactg
atgcacaacgccatctgtttgcaaataaactttctgaacttcctgaaatgggtcgttattcccaggga
actgaaagctatccgcaatttgctattcgtattgctgagatgctgcaagaccctgaccgaataaaaga
actatacccatacctaaaaaaagtgggatatatgccatcaaataaaaaggacaccgtaaatggctaa

> repAci3_p203_GU978997
atgaaaacagaactaatagttaaagataatgcattaattaatgccagttataaccttgatctagtcga
gcaacggttaattcttctagctatcgttgaagcaagggaatcgggtaagggaataaatgctaatgatc
cattaacagttcatgctgaaagctacatcaatcaatttggtgtacatcgaaacacggcttatcaggca
ttaaaagatgcttgtgatgatctatttgcaagacaattcagttatcaaagtcttagtgaaaaaggcaa
cattattaatcacaagtcaagatgggtgagtgaagtcgcttatattgataatgaagcggttgttagac
ttatttttgctcccgctatcgtgcctttaattacaagactagaagaacaatttacaaagtatgaaata
caacaaataagcaatttaacaagtgcttatgccgttcggttatatgagatattgattgcatggcgtag
tacaggaaaaacgcctcttataactttgtctgatttcagacaaaaaataggtgtactcgatactgaat
acaaacgaatgtatgactttaaaaaatatgtcttagacattgcattaaagcaagtaaatgaacatacc
gacattactgttaaagttgaacagcataagactggtagatcaattacaggtttttcatttagctttaa
acaaaaaaaatcagtgacgaagtcagctaaaagtataggtgtaagcgaagatataacgatcactttaa
cagatgcacaacgctattcatttgcgagtaaattgtcagagcttccagaaatgggaaaactttcacaa
ggcaccgaaagctatgaacaatttgctgtacggattgcagatatgctaaaacagccggagaaattaaa
agaacttactccattacttcgaaaagttggctttcaataa

> repAci4_p844_GU978998
atatgactacgtttacctaccaatatgactacgtttacctaccaatatgactacgtttacctaccaat
atgactacgtttacctaccataagtctaccttatttattttgtagatttaaccatgtttaccttgcat
taatctacagatttattaatatagatttatattcataactaataagatagataaataaagtgcgggat
ttagtagttaaagataatgcattaattaatgcgagttataacttagatttagtagaacagcgtttaat
cttattagctattgttgaagcaagagatagtggtcgaggcattaatgccaatgatccattagaagtcc

atgctgagagctatgtaaatcaatttaatgttgcaagacaaacagcgtaccaagcgttaaaagatgct
tgtaaagatctattcgtacgccaatttagctatcaagaaatcaataagagaggaaatgtagaaaatgt
tttaagccgctgggtcagcgagattagatatatcgatgatgaagcaaccgtgaagttaatatttgctc
ctgcaattgtcccacttattacacgtttagaagagcaatttactaaatatgaattacaacaaattagt
aatctcagcagtgcgtatgctgtgcggttatatgaattgttaatagcttggcgcagtacaggccaaac
tcctattattgaactagcagagttcaggaaaaaaataggtgttcttgatgatgaatatacaagaatgg
ggaatttcaaagaccgagtattaaatttggctattgctcaaattaatgaacatacagatattaaagtc
caatgtcagcaacataaaaagggacgtaatatttctggcttttcatttacctttaaacagaaaaaggt
cgttatagctaacaataaaaagcaaactactcttgagattttctcaaaatttactgatgcacagcgac
atttttttgctaataagctatctgagcttccagaaatgaataaatattctcaaggtactgaaagctat
tcgcagttcgcagttcgaatttctgaaatgctaaaagatctacaaaaatttgaagaactactgccata
tctagagaaagtaggctttaatgcaaaataa

> repAci5_p537_GU978999
atatgactacgtttacctaccaatagactacgtttacctaccaatatgactacgtttacctaccaata
tgactacgtttacctaccataagtctacttattttttttttgtagatttaactacgtttccttgccttt
actacataatatttatatagatttatattcagaaataaaaaagtagactaataacatgcgagatcta
gttgtaaaagataatgctttaattaacgcaagctataacctagatctagttgaacagcgacttattct
tttagctattgttgaagcaagagaaagtggtaaaggcattaatgcaaacaacccctttagaggtgcatg
cagagagctatatcaatcaattcaatgttgcaagacagactgcctatcaagcattaaaggatgcttca
aaagatttatttgctagacaatttagctatcaagagatgaataaacgaggaaacatcgaaaacgtact
aagccgatgggttagtgagattcgttatattgatgctgaagcgactgttaagttaatttttgcacccg
ctattgttccattaattactaaactcgaagaacagttcactaagtatgaattacagcaagttagtaat
ctcagtagtgcttatgctgtacgcctatatgaattattgatcgcatggcgtagcactggccaaactcc
tgttatagagcttgaagagtttagaaaaaaaattggtgtgcttgatgatgagtacacaagaatgggga
actttaaagacagagtcttacatctagctatagatcaagttaatgagtttacagatatcactgttaaa
tatgagcagcataaaaaaggacgttcaatttatggcttttcattctcattcaagcaaaagaaaaacgt
taacaaaccaaatctagaagctagagatcaaaacaccttagatattttcaccaagttaacagatgccc
agcgtcatttatttgctaacaaattgtcagaactgcctgaaatgagtaagtattctcaaggcaccgaa
agctatccgcaatttgccgtacgaattgccgaaatgctattagatgctgaaaaatttaaagaactata
tccatatctagtaaaggttggctttcaaacaaaataa

> repAci6_pA85-3_KJ493819
atgggcatacttatgactgtaaactctgttaatttaaattctaaaaaagactttataatcaatagatt
atatgaaaacctaccaaaaaaaccttactgtaccagtgacttcttcggattaaagattcgtgacaaga
aacaagctatacgtcactcacatatacaaattaatcatccgaattttaaaagatatatagttattgat
gctgattatccaggcgcagcaacagcttggcggtatgattttgatgacaatattccagtaccaaattt
aattgtagttaatccagaaaatactcattgccatttttactatgaacttgaagctccagtaagtttta
ctgaaagttccagcaagagagctcaagaattttataattcagtttctaaaaagctcactgaagtatta
aaaggtgacagcaagtacgtgggactaatagcaaagaatcctgcacatgaaaaatggattgtagaagt
accacgattagaaaagtattcattgcatgaacttgttgaacatttagaacttaagccacatgaataca
gaaatataaattcagaaaaaaccggtatagagaaatttgtaattaacggccgtaatgatcatctattt
aacgaaatacggcatcaagcatatatcgacattagaagttatagaagtaaaacatttgttgagtggtt
tgatcatgtaaaaagcttattaataaatgcaaataagaattttagcgttccgctaccatattcagaag
tttgtgctacagcaaaatcaatcgcaaaatattgttggaaaaaagatagctactgctttcaagagttt
tgtgaaaggcaacatattaaagcaaagaaaggtggacgtgcaaaatccgataaatatgttgaaatgag
gcgcacggctgcgcgtttattgcgttctggtaaaactaaaacctatatatcagaattacttcaagtat
cgtacagaagcgttctccgttggcttcaaggcataaaagtacaagctgcaataatgcatttatcagaa
ttaaaaaagttatgtgacaatgcccaaaatcagatattagcctgcttcattgcttctctaatcgttat
tattttagatgagcacatttatgattttaatgaatctgaagaattaaaaattaccatcaaattcaaat
taaaagttccaattcaataa

> repAci7_p736_GU978996
taaaacgaggtttaccttgcattaaaacgaggtttaccttgcattaaaacgaggtttaccttgcatta

aaacgaggtttaccttgcattaagcgagttaataatataacctcgtcttattaaattaaaaacataat
cttttcatatgaaaacagaactaatagttaaagataatgccttaattaatgccagttataaccttgat
ctagtggagcaacggttaattcttttagcgatccttgaagcaagggaatcgggtaaaggaataaatgc
taatgatcctttaacagttcatgctgaaagttatatcaatcaatttggtgttcatcgaaatacggctt
atcaagcattaaaagatgcttgtgatgatctattcgtaagacaatttagttatcaaagccttagtgaa
aaaggaaatgttattaatcacaaatcaagatgggtgagtgaggttgcttatattgataacgaggctgt
cgttagacttatctttgctcccgctattgtgcctttaattactagactagaagaacaatttacaaagt
atgaaatacaacaaataagcaatttaacaagtgcttatgccgttcgtttatatgaaatattaattgca
tggcgtagtaccggaaaaacgcctctcataaccctgtacgacttcagacaaaaaataggtgtactcga
tactgaatacaaacgaatgtatgattttaaaaaatatgtcttggacattgcattaaaacaagtcaatg
aacataccgatattactgtcaaagttgaacagcataagacgggcagatcaattactggcttttcattt
agctttaaacaaaaaaagtcagctactcagtctgtcggatctaaaagagatccaaatacattggaccc
tttttcaacaatgacagataaacaacgtcatctattcgctagtaaactctccgagcttcctgagatga
gtaaatattcacaaggtacggaaagctatcagcagtttgctgtacgtatcgctggcatgctgcaagat
acagagcgatttagggaaattaattcctttgctcaaaaaaagtaa

> repAci8_p11921_GU979000
taggtttatcgacccataaaattaggtttatcgacccataaaattaggtttatcgactcataaaacta
ggtttatcgaccttaaaactaggtttatcgaccttaaaactaggttgatttacaagtccattaaaata
gtctattgtacgacctagtttaattgtaaatttattattagaaatgagtgaattaatcgtaaaggata
atgctttaattcaggctagctatactttagatacagttgaacaaagactgatcttattagctattgct
gaagctcgagaaacaggacatgggataaatgaaaatagccttctacaagtacatgcaagtagctatat
aaatacctttaatgtcgagaaacatactgcctataccgtacttcgagatgcatctaaaagcttatttg
atcgctatgtcacataccatgatattaatcctaagactgataaagaccgtagctttcactgccgctgg
gtcgacaaaattggatatgaacctcaatccggaatcgttttcctacgatttacacaagacattgttcc
actcataactcgtctggaagaaaatttcacaaaatatgaactgcagcaggtttcaaggttaactagct
cttacgctattcggttatacgagttattaattcaatggggatctcgagggaaaactccaacttttgat
ttacatgtctttagaaaccgacttggtgttgaagatgggcaatataagactatgtgcaattttaaaca
atttgtcttagattttgctttaaaacaaattaatcaatttacagacatcatagcgaaatatgaacagc
ataaatctggacgaaaaattacaggctttagctttacctttaaatttaaaaataataaaaacgtaaaa
gaaaaattagttgaaaaaactgagttttataagctcactgaatcgcaactagacctatttgcaaaaaa
gctagcgcatttacccgaacttggacatttagcagacgaaggtatgtcttatgaggaattttattcta
aattaaaaagcattttaaaagatccagaacagcaaaaaaaattagttccctatttgaaaaagcggga
ttaaatcctaaataa

> repM–Aci9_pMAC_AY541809
atgagtgaattaatcgtaaaggataatgctctaattcaagcaagttataccttagatacagttgaaca
aagactgatcctattagccattgctgaagctcgagaaactggacatgggataactgaaaatagtcttt
tagaagtacatgcaagtagttatataaatactttaatgtcgagaaacataccgcttataccgtactc
agagaggcatctaaaagcttatttgatcgctatgtcacataccatgacattaatcctaagacaggtaa
ggatcgtagctttcattgtcgttgggtcgacaaaattgggtatgaatctcaatcaggaattattttcc
tacgattcacccaagatatcgttccactcataactcgacttgaagaaaatttcactaaatatgaattg
cagcaggtttctaggttaagtagctcatatgctattcggctatacgagctattaattcaatggagatc
tgccggaaaaacgccacttttgatctatctatctttagacaacaacttggtgtcaaacctcatcaat
acaaaacaatgagtaactttaaaacatatgttttagattttgctcttaagcaggtaaatgagttaacc
gatataacagctaaatatgagcaacataaaaaagggcgttctatttcaggtttttcattcactttcaa
acagaaaaaaatgagtaatctgccaataaaaaataagcgtgacccagacactatagatattttctcaa
aaatgacagatgctcaacgccatctgttttcccacaaactgtcagaacttcctgaaatgggaaagtat
tctcatggtacagaaagctatccgcaattcgctgtacgcattgcagaaatgcttcaaaacccagaaaa
gtttaaagaactctatccttatctccaaaaagtaggcttcaaagctgcatag

> repAciX_pACICU1_CP000864
atgaaaactgaactagtagttaaagataatgctcttattaatgcttcttataatttagagcttgcaga
acaaagactaattttactttccattgtaaaagctcgggaaacagggcgagggattacttctgacagtc

gtttagaggttcatgctagtgactatatgaagcagtttaacgtggagaaaagtgcagcttatgaagta
ttaaagagcgcatcagaaagtctctttaatcgttacttttcctataaggaacaaagacatgatggaac
ggaatttgtggttaaatcacgttgggtcagtcgtgtagcatatgctcccaatgtagctatattggaag
taacttttgcaccagatgttgtgccattaattactcgattagagcagcatttcactagctaccaacta
aagcaagtttctcaacttacaagtaaatacgctattagattatatgagatgctgattgcatggcgtaa
tgtaggaaaatgttcttttgagctcatcaatttgagagatagcttagggattgcatctgatgagtata
agcaaatggggcatttcaaaagtcgtgttctagacgcatcaattgctcaaattaatgagtacacagac
atcaaagtgacttatgaacaacagaagaatggtcgaactattagtggattcaccttcaagttaaagcc
taaacaagtacaacaagaaattaccatcctagatacaaaagcatcgttaattccttctgatctgactc
caaaccaacgtgttactttgctagtaaattatcaaaactccaagaattaggaggaaaagcagaacct
ggtgaggaagtagaggcatttgcaaaacgaattgagttatggctagaggatgaaaaaaaattgaagat
gcttactccatttctctatcaagtaggatttaaaaaagctaaaccaaagaaagtatctcagtag

> p3ABAYE0002_p3ABAYE_CU459140
atgagcaatcacaacgaaccagaagaattagaacatctaccttattgtattggtaacattcgacataa
tggagtagtcagcactagtaaccgtctgattagaccaattgaattatcatctaatgagtataaagctc
ttctttatgcaatggctgttgcgaattatggtgagaagaataatcaagatcgtgaaattacagagcaa
acttatatttatttgcataaagatgatttaggcgaattattaggattagataaaaagaattccattaa
tgttgctattgatcgtatttataaagaattatcatcacgagtagctcatttttgtaattgaagagcccg
tggatgataataagcgtaaagttaaaaaagtacactcagtagtaccaattattcgtgaactccgttgg
gaagatgatgctaaaaatgctttgcagatacgtttcaccagtgaagtacttccgtattttactcgttt
agcaaatggaaattttacgacctatcaattgaaagatttatttgctttggactctgtaaccagtatga
gtttgtattcatactttatcaaaaatgaatttaagtatgctaatcaagattcttatgaggttgaactt
tcacttgagaatatcaaagcattaattgatattggggagaaaaaatacgatcgttgggttgattttag
acgatacgttttagacaagattattgaagaaattaatgaaaggactagtcttcaattagagtacgata
ccatcaagaaaggtcgacctattgtcggagttcgatttaaaattcttaataggcatgccactgaagtt
gttgtatctaatactaatgataagactcaaatctatttagatgtcaattttgatgataacgcgcttgt
taaagaattaggtgcgaagttcgatatgactgtaaggtcttggtacatctatgcaaacgacccaaatt
ataaacagtttagtaagtggtttaagacagaagggtgcttaactgaatctcaagcaaatgtgatagtt
aatgatattatgttccaaatggattttgctgtagctggcagttcaatgagtgaatttaagaaagaaat
gaaatataaattaaaaaataatccaaattttgtaaaagaaaatagaaaacgattgaatgaaatctttg
gaaaagacgttatatga

> p4ABAYE0001_p4ABAYE_CU459139
ttgcagaatttagataagaaaaaacccctgttatcggacagtttggcgaccggtgataacaagggttt
tgcatctcccaaaggagatcaacatagggatagaataacacgttttggcattttgaaacatagatcga
agcaacaagaaaactatttattttcgttagctaagattaaagaaaattatcatgccgatgtaaaaaac
gatgaatctattcgcgccatgaaaactgcccaaaaattaaatgggtgcggtaattttcttctattcaa
aaatttttacaccattaatcaaattaaactcgccaagttccaagcttgtagtgagcatttgttatgtc
cgttttgtgctggtattagagcttctaaggcaattcaaaaatactctgagcgtgttgatcaagtctta
tctgaaaatcctcgtttaaagcccgttatgatcacgtttacggttaaaaatggggtagacctaggga
acggttcacccatcttataaaatcgtttagaacgcttatagagcgtcgtagggactatattaaaaaag
ggcgtggctttaatgaattttgcaaaattaatggtgcgatgtattcatatgagaatacttacaatgaa
aaaactaatgaatggcatcctcatattcatatgtttgcacttttggatgattggatagatcaggatga
attgtctcaatattggcaatccattactggggactctatggtcgttgatattcgtagagccaaaaaac
aaaaagacttaggctattcaggtgctgctgctgaagtctgtaaatatgctctcaaatttggtgatctt
tctgtagaaaagacttgggaagctttcaaagttttgaaaggtaagcgattaagtggggcttttggatc
tctttggggcgtgaaaattcctgaatcattgatagatgatcttccagacgattctgatttaccttatt
tagaaatgatttataagttcgtcttttctaagaagtcttattacgatttacaacttactcgtcatgtc
gaacctacaggtaaggacgacgccgacgagcttcgaggagaagaaggacgcaacctgttggtgagcat
ggacgggcgaggagcgagcgacgctgggagggcccgcactggcgcgctagccccgcagcacggacgaa
aaaaacaacactggcaaattccaccagttactcgtgttcgggttcggaagcgaatccgaagatgggac
ggatatttatgtgtcttacatttatag

> RepApAB49_pAB49_L77992
atgtcgatgaatatattttatggggaaaatccgtcctcagcaagcttaaagagcctcgaaaatagcca
gtcccttggtataaagacgaaatctcatgtaatgcttacgccacaaggctttcagcgtgtgcatgatt
atctattacaagaccaatctagaaaacttcttcctaaagaacgtgtatctaaatgtagacgtctccgg
atcgataaaactaagactagaactgttatgtataacgagcatcgagagaaggctcattatggcaatgt
tcaaatctgcggttctatttggtcatgtcctgtttgtgccaagcaaatcacacagaaaagacgtaatg
aattaggtaagggcatagagtcgtggaaaacggttcataatggctctgtatatctccttacgcttact
tttagccattcacctgaccaatccctcaaaagtaatttagagggccttaaacgcgcaatgaagcgttt
ttatgagacaactcgagttcaggctatttttaaaaaactatctgtttttcacaaaataaaaggcctag
aagttacatacggtcagaatggttggcatccccatcatcatgtacttcttttagctgaacatcatgat
ttacgttttaaagattacacttctgaattaacggagttatggattaaagcctgtattaaatcaggatt
aaatgctccatcgatgcgccacggtttagatcttcgaaatggctcttatgctgaccaatatgtgtcta
agtggggccttgaagatgaactttcgaaagggcatgtgaaaaaaggtcgcaatggtggttttacccct
tttgatcttttaaatttttctattgaagataatgaaatttatggaaaaaaaccttctaaacttttcca
agaatttgccatttctatgaagggtgctcgccaactagtttggtctcgaggtcttaaaaaacttttag
gtattgaagaaaaaagtgacgaggaacttgcagtagaaactgacaaagcttccattactttgaatcgt
gttgaagatctcgtttttgaacttttatgtcgttatcaattgcgtcatcaatatcttgaagcaattaa
acatgattatgagactggctcttttggttctggattagcagatcaacttattgagcaagtagtgaatt
atgaaattaaacaaatgcagcaggtattttcgtga

> p1ABAYE0001_p1ABAYE_CU459137
atgaataaagaaaatagttatgataaatcttatccagtaacaacaatggctattcaaaacaaagttac
tgaatgctttaaaagcatgtctgtagatgaaaaaagaattttaattatggcttctccgattgctagaa
atgtcgatgcaagtgaacaagatcaaatcttaatatctgctcaacaatttgctgatgactgtggtatc
aaagtcaattctgcttataaacaaattgaaaatgcgtcaaaaaaactagtagatcggtccttttcata
cgttaatgatagggggaaaaaggtctactctaactgggtaattgatgctacttatgaagatgcaggga
tatctttaaggtttacatctattgttttggtgatgttgaaaattttagataaatacaatccatacact
cgttataaaaaagatgtagttctaaaattaaaaaaagactactcaatagacttttaccatttagcgaa
aaaaaatcaggcaaaaaatggctttgaattaacgctagatgaaatgtttacagagtttggtttaccag
aatcttatagagatttgaggaacttaaaacgaagagttttgaagagctcattagatgaaattaatgaa
tttaccgatgtaacagttgactatagtccagttaaaaaaggacgttctgttgtcggctttaagttcac
tgtgaaagaaaaaatctaagccaaaattaatagctcctgagcgagatccaaaaacaatagatatgttct
gcaacctgtctgatgctcaaattaacaagtacagtgctattttatctaaactttctgagctatcagac
ctaagtaacttccaggactatcctagttttgccctttggattagtggcatcttacgagatccgaaaag
tgtaagagaggaaacagcaaagcggatttttaaagctcttcatagcaagacggattttaaaccatga

> pD46-4_CP012956
atgacagaatctgaaccattgcaaaaagaatcgaattatattccttactgtataggcaacattcgcca
taatggtgttgtcagtaccagtaaccgtttaattcgcccaatcgagttatcagccaatgaatacaaag
cgcttctatatgcaatggcagttgcgaattatagtgagaaaaatagggtaaatggtgaaatcaccgaa
caaacctatatttatttgtataaggatgatttggcagatttactaggattgaataaacgtaattcaat
taatgttgcaatagaccgcatctataaagaactgtcatcccgggtggctcattttattattgaggagc
ctgctgatgatggtaagagaaaaactaaaaaagtacattctgtcgttcccattatccgtgagttaaga
tgggaagatgactctaaaaatgcgattcagattcgttttaccaatgaggtattgccgtatttcactca
actggcggggggggaattttaccacctatcagctaaaacatctttttgccttggattctgttgccagta
tgagtctttatacgtatttcattaaaaacgaattcaagtacgccaatcaaaagagttatgaagtgcca
ttacttttggaaaacttgaaagcagtgattgatattaatgaaaccaaatatgatcgctgggtagattt
tagacgctatgtattagacaaaatagtggcagaaattaatgaaaatacagacctacaattagaatatg
aaaccgttaagaaaggccgtccaattgtgggtgtgaattttaaactgcatcatcggattgctgataaa
gcaccggatgaaattgcagtaattgaaaaaatctatcttgatgttccatttgaagacaatgcattcgt
aaaagaattgggggcgaaattcgatacgaatatacgttcttggtatatttttaataatcatgaaaatt
atcagcaattcaagaagtggtttaaaaaagtaggatgtctcactgactctcaagcaaatatcgttatt
aatgacactttattccaaatggattttgctgaaattggtatgggtctgaatgactttaaacgaaatat
gaagcataaacttaaaaataatccggaattcgttcaaagtattcgtgaaagactgaatgatatttttg

ggaaagagcttatttag

> pABTJ1_CP003501
atgataactagcaatatctttgctactgataataaatctatattaatcagtaggttgtatgaaaatct
accacgaaaaccatattgtaccaatgatttctttgggttacggattcgtgaaaaaaaatctgcaatat
cacactcgcacatacaatttaatcacccaagctttaaacgctacatcgtaattgatgctgattatgct
ggggctgctacagcttggcgctatgaattcgcagaaaatatacctgttccaaatctaatagtcacaaa
tcctgaaaacagtcactgccatttttattatgaattaagtgctccagtaagttttacagactcatcga
gtaaaaaagcacaagaattttataatgctgttagtaaaaagcttactgaagtactaagaggggataca
aattacacaggactgatagcaaaaaccctgctcatgaaaagtggattgttgaagcccctagaattga
aacatacagcctacatgagcttgtagagcatttagagctacatcctcacgaatatcggtcaataccag
gtcaaaatagcaagcaagagcaagtacaatgtataaatggccgcaatgaccatctttttcatacagta
agagttaaagcctatgttgatgttagagacttcagatcaaaaacctatccacaatgggaagaacatgt
aagacaattgctggtagatcataatttagagttaaataacccattaccttattcagaaattaaagcaa
cggccaagtcgatagcaaaatattgttggaaaaaagacggctattgctatcaagagttttgtgatcgc
caaatatctaaagcaaagaaaggtggtcaagctaaagcagataaatatatagaattaagaaaaaaagc
agtggctttgcttagaaaggggaaaaataaaagattaatagctcaacttctaaaagtttcctacagat
cagttctacgttggctttataacgtcaaactagcggcggccataatgcatcttagagacttaaagaac
atgtgtgacaatgcccaaaatcagatattagccgcttttgttgccagcttagctgtcttattccttga
tgagtttatttatgactttactcaagatgatattttaacaattaacttaacttttaatattaagatgc
ttatttag

> pRCH51-3_KY216144
atgcagaaaattattggctcacaaggcttagaggactatacacaatctaatacaagggcaaaattgac
cgatttagttgtgacccgcaatgattttcctactgccagatatagcattgatctcaatcttgagaagc
ttatgtactgtgcaatgatcatcgtgagaaagaatgagcttaaaaataagacctcaattactcatgac
gacttcatttatgtgagcagcgaaaactttggagaattgacctccccaatggctagaaaggaagtgct
tactgcaacagataaacgtgaaattcagcgaaatgctgaaactgctttaaaacgtatttatacaaaat
ttgataacccaactatgttggttaaagatggggaatctgacgaacctgctaaagttccgatgatgact
tattgccattatgacaaagcaactaaatgtattaaggttagattcgcaaaagaatttttttgagtattt
ctatgatctagttaagaaagtagatgaaaaaactaagtcatttagtagccatgaactgaagcatatta
tcttatttaattcgagctattcccttcgtttgtatcggatcttaatgagctatatgtggcgtacatca
gaggtcactattgatctggaagaattaagatggatgcttgaatgtgaggataaatataaagagttggc
caattttaaaaaccgtgttttgaatgtggctcaggatgaaattaacgaacttagcaatattaatgtca
gttttgagaacgtaaaaaacggaaaggaagtagttgctattaagtttatttttagcatgaaaactgaa
tataaagagcaaggacacatcaaatttatagataaaatgaaaaagggctatctggctgctgcaattcc
atttagtgatgatggatcacactttaaagcacctgatcgtattaagcatttcaaaccaccagtaaagg
tatctccaaaacaaatcagcaccttagtaaattgcaaagaatttttaaatgattacggatatttctta
ggtaatctagacgaggatacttctaaggtaatcatgagaactctattaactgaaaagttagataagct
taatgctcataagccgatagatatggattattacttctggttacaggcaaaacgagggattatcacca
atagcaataatgataacaagaacgatcaggacacagacaatcaggacacggacgatcaggattaa

> repA_AB_pABIR_EU294228
ttggataagaatcaaattgttaaatcaaatcaagttatagaagcttcctatcaattaagtgctgtaga
acagcgtatcgtcttggcagctatttcacgtattccgaagagccagcctattactgatgatgagttat
accctgtaagcgttaatgaactacagctattaggagtacatgaaaaaaccgcatacagagacttaaaa
gaaggtattaatagactttatgaaagatctattaatcttagtattgatgataaatctataaaaatgag
gtgggtacaagagatccagtttttggatagtcaaagtatcattggtattcgtttttcaaaacctatct
tgcccttcatatctaatttaagtagagaatttactaagtatgctctatcagatattgctgggatcaat
agtggatatggtattcgtatttatgaattattggtgcaatacagacaaataggtaagcgtgaaatctc
tgttgagagcttacgaagcatgttggagcttggcaaaaaatacccactatttgcagatttcaaaaaac
gggtaattgatactgctgtagaccaaataaatgaatgtagcccattaagcgtttcctatgagcagaaa
aaaactggtcgtaaagtcacccatattctttttcatttaaagaaaaatcaaaaagcattaatcagca
gaacgaacaagataaggtttataaactaacagacgctcaaatcaatctgtttggcaatcagctttctc

gcttacatgagctatcgcatttagcagctcaaggagaaagctatgatgtactggcatcaagaataaaa
gaaatgcttagagatccaatacaacaaaagcaatttattcctcatcttaggaatttaggttttaaagg
gtga

> pA297-3_parA_KU744946
atgaacgacaagacttcacaaattaaagatgccggtatcttaactattggtaatggaaaaggtggcgt
cggtaaaagtacatttaccgtacattctgcgtactttttttctcaacatggctacaaggtaattgtcg
tggaccttgataatcaaggcaacagctctggtcatttattaatgctcaattcaaatacagtattacca
gaatatgtgacgcgaccaacctcacctgtacataacttaggggacgcaatttctctttttcttaaaga
taatcataatcttaagtgcgacttacaaaatggctcaattggcgtttttcgaccaactaaagcactcg
ctaatataaacacgagtagcgatgttgatctagttactatttttaaagataatattagtaagttattg
aaatataaaacagttattatattcgacacacccccctactctaagtaatttaatgcttctacctttatc
agtatcaaatgtagttttaattccgacaatgcttaaacaatacgcatcggatggtattcgagattatt
tgtcttttgctaataaaattaaaaaaacgaacaaccccaagttattaattggcggtattgttcccaat
atggttaatttaaaatcaaatattcaaaagaatgaactatctaagttgttggaagtggctgagcaaac
caatttgttctaccaagataaatctaatatggcatatataacggataagaatgttatagaagaagcag
tcacttatggcactgcttgttggcatattaataaaacatcatcacgtgatgtcaaacgaacatttacc
aatatattcaaacaagttggcactcaacttaaattatctgtgttcaatacatctggtaaaggagaata
a

> pRAY*_unknownorf_JQ904627
ggatcccgcctacgatcatgttcattcaaaaatataaaaattgttttatctgatttttcaggagtaat
cttaaataagaataggtttacgaatgttcacttcaaaaagtgcgtattttatgctgctatttttagag
attgtcgctttaaaaattgcacatttgaaaaatgcatttttattaatacaaatacagaaagcctaaat
cactcgatattagactgttctaaaaaatataatttttacgaaatgaaaattcctgatgacttaaaaga
tcaactaacagaatatagaaatattccaatactgcaaaaaaaccgtttacttcatctaaaggggggaa
gaatcaataccgcaaccttattcattctcctatccaaaatatccaaatacaagctgctaaatggtctt
aaaaaaatttatattgaggaacaaagtaaagtaaaaatacttagcactttcaaactccttgaagaaat
aaagaacctagttgacactaacaccgcattcaaacagaataatggtgctctgccccatccaatcgaaa
ggttggatagttaa

> pS30-1_KY617771
atgaaaaccgaactaattgtcaaagataacgctttaattaatgctagttataaccttgatctagtaga
acaacgattaattcttttagctatcgttgaggccagggaatcaggtaaagggataaatgctaatgatc
cattaacagttcatgctgaaagttatatcaatcaatttggtgtacatcgaaatactgcttatcaagcc
ttaaaagatgcttgcgatgacctatttgcaagacaattcagttatcaaagtcttagtgaaaaaggtaa
tgtcattaatcacaagtcaagatgggtaagcgaggttgcttatattgataatgaggctgtcgttagac
ttatttttgctcctgctattgtgcctttaataactcgattagaagaacaatttacaaagtatgagata
cagcaaataagcaatttaacgagtgcttatgctgttcgcttatatgaaatattgattgcgtggcgtag
taccggaaaaacgcctcttataactatctacgatttcagacaaaaaataggtgtactcgagactgaat
acaaaaggatgtacgattttaagaaatatgttttagacattgcattaaagcaagtaaatgaacatacc
gatattaatgtcaaagttgaacaacataaaaccggcagatccattacgggcttttcatttagcttcaa
acagaaaaagtcagcaacaaatacagccaatgacattagtcaggacaaagagctaaaaatcaatttga
ctgatgcacaacgatatttatttgctagtaaattatcagaacttcctgagatggctaaactttcacaa
ggtaatgaaagctatgaacagtttgctgctcggattgtaaccatgctacaagagccaaataaattaaa
agaatttatgccattacttcgaaaagtcggctttcaatga

> pABTJ2_NC_020524
ATGGCAGAACTTATTAGGAATTCAGATGTTTATAAAGCGAATGCATTGATTAATGCAAGCTACGCTTT
GGACACTGCTGAGCAAAGAATAATTCTACTCGCCATTTTAGTTTCCAGAAACAAGAATGCAGATCTGA
CTGCCGAAACGATTATCGAGATTCCTGCTTCCTTATATGCCCAAAAATTTAATACAACAGTGAGCGCG
GCATATAAAACACTGAAGGAAGCCGAAGATACCTTATTTGAAAGACGTTTTTCTTACACCACAATGCG
AAATGGCAAGATTGAGGTGGTTCGGTCACGTTGGGTATCACGAGTTTCATACGTTAAGGATGATGCAT
TATTAACGATCACCCTAGCTCCTGATGTGATTCCTCTAGTAACCAAGCTAGAAGGAACCTTTACCAAA

TATGCCATCGACAATTTACGCGATGTGACCAGTAAATATGGCATCCGTCTGTATGAGTTGGTCGCTAG
TTGGAAAAATTCGGATATACGTAAAACTCCTGTTTATGACTTCGAGGACTTCCGTGCCAAGATGGGCC
TTCTTCCTCATGAGTATAGAGACAAGAAAAATCCCGAAAGTACGGATATGACCAACTTCAATAAACGT
GTATTGAAGCCGGCAATTGATCAGATTAATAGTTTTACTGACCTGTTTATTACTGAAAAGAAAATCAA
GACAGGACGTAATATCACGGGCATTTATTTTGAAGTAAGTTTAAAAACCGATAACTTCATTGAAGGCG
AAGCGAAAGAAATCCATGACAGCAAGCCTTCTTCCGATTCAGCTAAAAAGACAGGCACTCCTTTAGAA
AACATTAGGCTTCCAAAAGTATCGACCCAAGAGTTTTTGGGTAGTGATCTCAGCGAAGAGGACCTTAA
CAAAGAGAACCCATTAAAAGAATTTATCGTTGAATCTGGCGTTTATAAGTCTGCTATTGAAAAGCCAG
TAGAAGAAAAGGATGAATTTGAATTAAATGGCATAAAACGATTGTATGAGGCTCTATTAAAGCTGGAT
GAAGGCGTAACCAAGGAATACGTCCGCGAATATGCCCAGATTAAAGGCGTAACTCTACAACACGCATT
AATTGAACTTTATAACTCTAAAAGACCGGCTTAA

> pXBB1-2_CP010353
ATGAAGAATGGTTTAGTTGTGAAAGATAATGCATTAATAAACGCCAGTTATAATTTAGAACTAACAGA
ACAGCGTTTAGTCATGCTTGCAATCATTAATGCAAGAGAATTAGGGCAGGGTATTACGGCAGATAGCA
AACTAGAAATACATGCTAGTGACTATGCAAAGCTATTTAATGTATCACCTGATGCATCCTACAAAGCC
CTGAAAGATGCTGTAAATAATCTATTTAACAGGCAGTTTAGCTATACAGCCGAATATAAAAAAACAGG
GAAGGTAGGTATCGTTCGCTCTCGCTGGGTGAGTCGTATTTTTTATGTAGATGATTTGGCATTACTAG
AAATTACTTTCGCACCTGATGTAGTGCCTTTAATCACTAGATTAGAAGAACACTTTACAAAATATGAA
GCCAAACAAGTTGCACACCTCACCAGTAAGTATGCCACTAGACTTTATGAGTTATTAATCGCTTGGCG
TGAAGTTGGTAAAACACCTGTTTTTGAACTACAACAATTACGTAAAAATTTAGGTGTTGAAGATGATG
AATATCAACGTATGCACCATTTTAAAAGCCGTGTATTAGAAACTGCCATTACTCAAATCAATGAACAT
ACAGACATAAAAGCCACTTATGAGCAACACAAGAAAGGGCGTACAATTACTGGGTTTTCATTCAAGTT
TAAGCAAAAAGTACAACCTAAAATTGAAACTAAACGAGATCCAAACACCCCTGACTTTTTTATAAAAA
TGACCGATGCGCAACGCCATTTATTTGCAAATAAAATGTCTGAAATGCCCGAAATGGGCAAGTATTCG
CAGGGTACTGAAAGCTATCAACAATTTGCTATACGCATCGCAGATATGTTGTTAGAGCCTGAAAAGTT
CCGAGAATTGTATCCTGTTTTAGAAAAAGTTGGTTTTCAGCCATAA

> pMS32-3_KJ616406
atgtcaaaagaattagttgtaaaaaccaatcggttaaaccaagcttttcaaactctatctttatcaga
gtttcatattgttcaattagccattgttgatgctagacatacgggtactggactaagtacagatactc
ctttacgaatagatgctttgagatatgctgaagtttttgggacaacaaggcaaaatgcttatcagaga
atgaaagaagcagaggattctttatttaacagaagatttagcttctttgatgaagatgggaaattagt
caaaagtagatggattcagcaagtaaaatatctagatgatgaaggagctattgaacttgttttcactt
tagctgttgtacaaggcataagtaaaattgatggtattaaagactttttttactcaatatttactaagc
caaacagcccagcttaacagtacatattcagcaagattatacgagttattaattcaatggagagctat
tggaaaaacgccagtatttgaattagcaacttttagagaacaacttggaattggtgttaatgaataca
agcgtatggatcactttaaaactagagttcttgacttagccatttctgaaattagcgaaaaaacagat
atagaggcaacctaccaacagcataaaaaaggccgttcaatatctggtttttctttttcttttaaaca
aaaaaaatctaaaactaaatcattagaaaatcagacaatatctgggaatctagatctttttttcaaaaa
aaatgactgactctcaacgccacttattttccaataagctatctgaacttcctgaaatgagtaaatac
tctcaaggaactgaaagctatcctcaattcgcagtaagaattgctgaaatgcttaaagattctgagaa
gttaaaagagtttgctccaatgcttgaaaaggttggctatcgttaa

> pNaval81-26_NZ_AFDB02000003.1
ATGACAAATACCAATAAGTTAGTTGTAAAGGATAATGCTCTTATTGATGCCTCTTTCAATTTATCCCT
AATAGAGCAACGGATTATGCTTCTAGCAATAGTTGAAGCTAGAGAATCAAACAGTCTTTCCCCAGATA
CCCCTATAGAAGTATCTGTGAGCGACTATATTCATCAATTTAAAGTGGATAGCAATAATGCTTATGCC
CTACTTAAAGATGCTTCTAAAACCTTAAAGCGAAGAGAGTTTAGCTATTTAGATAGATATAAGGGCAT
AGAAGCACTTTCAACAGCTAATTGGGTTAATAAAGTGACCTATGTTGATAAGAGCGGTTTGATTGTTT
TATATCTTAGTCATGAAGTAATTAGCTTAATTAGTAAATTAAGTGAACAATTCACAAAATACTATATC
GAGCAAGTTTCTGAATTTAAAAGCAAATATAGTATTCGATTATACGAACTTATTATTAAGTGGTTAAG
CGTTGCTAAGACTGAAAAATACAGTATCAATGATTTAAGATCAAAGCTTGGACTTGGTGTTGAAGAGT
ATTCAACTATGACCAATTTCAAGTCTAATGTATTAGACAAAGCAATTAATGAAATCAATAAACATACT

GATATAATTGTAGATTATCAGCAATTTAAGAAAGGAAGAGTGATTACTGATATCCAGTTCTTTATTAA
GTCTAAAGCTCGACCATCTAAACAAACTAACACTACCAAGCAATCTTTTTATCAAATGAATGATGCAC
AAATTAACTTATTTGGAAATCAGTTATCTCGTCTACATGAACTGTCTCATTTAGCAAATCAAGGTGAA
AGCTATGACGAGTTAGCGATTAAAATTAAAGATATGCTCAGAGATCCAATACAGCAAAAACAACTTCT
TCCACATCTTAAAAATCTAGGTTTTAAGGCTTAA

> pAB3_CP012005
gtgaagaagcctaagcatgacctgacccacgtccgacatgatcccgcgcactgtttggcacctggcct
gttccgcagcctcaagcgtggcgatcgcaaacgctgcaagctggacgtgacctacacctttggcgagg
acgaatccatgcgtttcgtcggattcgaacctctcggggccgatgatatgcgtcttttgcaaggcatc
gtggcccttggcggcccgaacggcatcttgctaaccccggaaccgaccagtgagacggggcgacagct
acggctattccttgaaccccgtttcgaagccattgagcaagacggcttggtggttcgtgagagcctga
ccaaactgctctcagaaacgggcatgacggatagcggcgacaacatcaaggcgctcaaagccagcctg
ctgcgcatgtcgaacgtcaccatccttgtgacgaagggacggcggcaagccgcgttccacctgatgag
tcatgcttttgacgagacggacggcaggctatgggttgccctgaatccgcgtattgccgaagcgatcc
tggggcatcgtccatatgcccgtatcgacatggcggaagtgcgggtgctacagactgatccggcacgg
ctgatgcaccaacggctatgcggctggatcgaccccggcaaatccgggcgcgtggaactggacacgct
ttgcggctatgtctggccagatgaagccaatgccgaagctatgaaaaaacgccgtcagactgcccgga
aggcactggccgaacttgccgccgtgggttgggtagtgaacgaatacgccaagggaaaatgggagatc
aagaggcctggccccacggcaactgcacccgtttaccgtcgtaacgttcccttgttaccgtcgtaa

> p1ABSDF_CU468231
ttgaaaaaaatatgtgtacttatgaagaaggaacttgttgtcaaagacaatgcactaataaatgccag
ttataatttagacctttcagaacaacgtctaatattgttagcaatccttgaagctagacaatcaaaca
cacccaatgataaagatttaacaattcatgctgaaagctatatcaaccattttaacgttcatagaaat
acagcctataaagtccttaaagatgcatgtaagagtctatttgatcgtagattcagctatcaaaaact
aactcagaagggcaacattgaaaatgtaataagccgatgggtacaacgcatatcttatgttgagaatg
aagctcttgttcgtattaagttttctgatgatgttgtaccgttgattacaaacttagaaaaacacttc
accagttatgaattagaacaagtcagtagtttaaccagtgtttacgctatacgcttatatgaattgct
tattgcatggcgtagtactggtaaagtcattttggtagagctagaagaacttagattaaaactaggta
tagaatcccatgaatataagagaatggggcaatttaaagaaaaagttttacaccttgctattgatcaa
ataaacaaatacaccgatataaaagcagagtatgaacaacacaaacgtggccgttcgattattggctt
ttcatttaagtttaaacagaaacaacaaccccaaaaagcagattccaagcgagcccctaacaccccag
acttctttgtcaaaatgaccgatgcacaacgccatctattcgccaataaaatgtctgagatgcctgaa
atgagcaaatattcacaaggcacagaaagctatcaacagtttgctatccgtatcgctgacatgctttt
agagcctgaaaagtttagagagctttatccaatcttagaaaaagcagggtttaaaggttaa
